# Resolving subcellular plant metabolism

**DOI:** 10.1111/tpj.14472

**Published:** 2019-09-25

**Authors:** Lisa Fürtauer, Lisa Küstner, Wolfram Weckwerth, Arnd G. Heyer, Thomas Nägele

**Affiliations:** ^1^ Department Biology I, Plant Evolutionary Cell Biology Ludwig‐Maximilians‐Universität München Planegg‐Martinsried Germany; ^2^ Department of Ecogenomics and Systems Biology University of Vienna Vienna Austria; ^3^ Department of Plant Biotechnology University of Stuttgart Institute of Biomaterials and Biomolecular Systems Stuttgart Germany; ^4^ Vienna Metabolomics Center University of Vienna Vienna Austria

**Keywords:** *Arabidopsis thaliana*, subcellular metabolism, proteomics, metabolomics, nonaqueous fractionation, hexokinase 1, photorespiration

## Abstract

Plant cells are characterized by a high degree of compartmentalization and a diverse proteome and metabolome. Only a very limited number of studies has addressed combined subcellular proteomics and metabolomics which strongly limits biochemical and physiological interpretation of large‐scale ’omics data. Our study presents a methodological combination of nonaqueous fractionation, shotgun proteomics, enzyme activities and metabolomics to reveal subcellular diurnal dynamics of plant metabolism. Subcellular marker protein sets were identified and enzymatically validated to resolve metabolism in a four‐compartment model comprising chloroplasts, cytosol, vacuole and mitochondria. These marker sets are now available for future studies that aim to monitor subcellular metabolome and proteome dynamics. Comparing subcellular dynamics in wild type plants and HXK1‐deficient *gin2‐1* mutants revealed a strong impact of HXK1 activity on metabolome dynamics in multiple compartments. Glucose accumulation in the cytosol of *gin2‐1* was accompanied by diminished vacuolar glucose levels. Subcellular dynamics of pyruvate, succinate and fumarate amounts were significantly affected in *gin2‐1* and coincided with differential mitochondrial proteome dynamics. Lowered mitochondrial glycine and serine amounts in *gin2‐1* together with reduced abundance of photorespiratory proteins indicated an effect of the *gin2‐1* mutation on photorespiratory capacity. Our findings highlight the necessity to resolve plant metabolism to a subcellular level to provide a causal relationship between metabolites, proteins and metabolic pathway regulation.

## Significance Statement

Our study resolves subcellular diurnal dynamics in the proteome and primary metabolome of *Arabidopsis thaliana*. These dynamics indicate a role for hexokinase 1 in photorespiration.

## Introduction

Regulation of biochemical pathways at the cellular level is strongly shaped by compartmentalization. While it is currently unknown how widespread compartmentalization is among prokaryotes, prokaryotic membrane organelles seem to have evolved independently (Diekmann and Pereira‐Leal, 2013), whereas eukaryotes are described to be derived from a highly compartmentalized last eukaryotic common ancestor (Field and Dacks, 2009). Furthermore, eukaryotic cells are characterized by the presence of mitochondria that have resulted from ancient endosymbiosis and whose acquisition is discussed to be the key step for a more complex eukaryotic cell plan (Diekmann and Pereira‐Leal, 2013). Among eukaryotes, higher plants show a particularly high degree of cellular compartmentalization due to the presence of compartments like vacuoles and plastids. Previous work provided evidence for an important role of cellular compartmentalization in stabilizing metabolism and showed that compartmental cell structure is a prerequisite for establishing stable metabolic states of different cell fates (Harrington *et al*., 2013). The detailed knowledge of subcellular biochemical networks immediately draws attention to the experimental resolution of subcellular metabolism. Therefore, the availability of experimental data on subcellular fluxes, protein levels and metabolite concentrations limits the applicability of genome‐scale metabolic networks to study organism‐environment interactions and the genotype−phenotype relationship.

Decades ago the method of nonaqueous fractionation (NAF) has been developed and applied to re‐calculate subcellular metabolite distributions in different organisms and tissues (Heber, 1957; Elbers *et al*., 1974; Gerhardt and Heldt, 1984). To date, NAF methodology has been applied in a broad context, for example analyzing compartmentation of primary metabolism in potato tubers (Farré *et al*., 2001), redox regulation of starch biosynthesis (Tiessen *et al*., 2002), stabilization of photosystem II by plastidial raffinose (Knaupp *et al*., 2011) or metabolic reprogramming of plant metabolism during cold acclimation (Fürtauer *et al*., 2016; Hoermiller *et al*., 2017). These approaches are based on separation of cellular compartments across a nonaqueous density gradient and the subsequent correlation of compartment‐specific marker enzyme activity distribution with metabolite abundance. This yields an estimation of relative subcellular metabolite distribution. For example, with such an approach it becomes possible to trace back cold‐induced sugar and amino acid dynamics in chloroplasts, the cytosol and vacuoles of plant leaf tissue (Nägele and Heyer, 2013; Fürtauer *et al*., 2016). However, the interpretation of subcellular metabolite dynamics revealed by this procedure is strongly limited due to several factors (Dietz, 2017). The omission of compartments together with the lack of robust and redundant marker sets belong to the most limiting factors. Quantification of marker enzyme activities in crude extracts of separated fractions is technically limited by appropriate (photometric) assays and their simultaneous applicability on the same fractionated sample. Consequently, replacing marker enzyme activities by marker protein abundance potentially increases both the number of separable compartments and the number of (reliable) marker proteins. Using such a strategy of combined NAF, proteome and metabolome analysis enabled separation of vacuolar, plastidial and cytosolic metabolites and proteins of Arabidopsis wild type leaf tissue, while mitochondrial and peroxisomal proteins clustered together with the cytosol (Arrivault *et al*., 2014). Observing that metabolites from different pathways, for example the Calvin−Benson cycle or glycolysis, grouped together with their associated proteins and the respective compartment provided strong evidence for the suitability of the NAF method to resolve metabolism in chloroplasts, vacuoles and the cytosol. However, limitations exist for the resolution of mitochondrial metabolism. Mitochondria represent one of the most distinctive attributes of eukaryotic cells (Diekmann and Pereira‐Leal, 2013) and are centrally involved in important cellular processes, for example energy metabolism, redox regulation, stress response and developmental reprogramming. Subcellular proteomics enable the detection and quantification of mitochondrial protein dynamics and, for example, its analysis in the context of respiratory metabolism (Lee *et al*., 2010).

In all phyla, hexokinases occur as enzymes of the glycolytic pathway, thereby contributing to breakdown of carbohydrates to provide carbon intermediates to numerous anabolic pathways as well as respiration. In different plant tissues, multiple hexokinase isoforms are found with different subcellular localization (Claeyssen and Rivoal, 2007). In Arabidopsis*,* Hexokinase 1 (HXK1) is located at the outer mitochondrial membrane, and it has been discussed whether it is translocated between mitochondrion and nucleus, for example via the cytoskeleton (Rolland *et al*., 2006; Claeyssen and Rivoal, 2007). In analogy to the observation in animals, it is speculated that a detachment of the membrane is induced by signals from G6P or methyl‐jasmonate (Xiang *et al*., 2011). Independently, HXK1 has a sensing and signalling function for glucose, besides its phosphorylating activity. The glucose sensing function integrates environmental, nutritional and hormonal cues in the metabolic signalling network that regulates growth and development of plants (Moore *et al*., 2003). The localization of hexokinase at the mitochondrial membrane was suggested to be a prerequisite for the signalling function (Xiao *et al*., 2000). The glucose insensitive mutant *gin2‐1* carries a point mutation in the HXK1 gene. This mutation has a broad impact, including alterations in vegetative and reproductive development, senescence, cell proliferation as well as gene expression (Moore *et al*., 2003). Sugars and sugar sensors have a broad impact on regulation of genes and influence several intracellular metabolic pathways from embryogenesis to senescence (Sheen, 2014; Li and Sheen, 2016; Aguilera‐Alvarado and Sánchez‐Nieto, 2017). Glucose seems to be one of the most ancient and conserved regulatory signals (Sheen, 2014).

The present study aimed to investigate the subcellular metabolic consequences of deficiency in glucose sensing and phosphorylation. A combination of NAF with proteomics and metabolomics analysis was applied to reveal compartment‐specific marker protein sets for a statistically robust estimation of dynamics of subcellular metabolite amounts. Compartment‐specific enzyme activities and liquid chromatography tandem mass spectrometry (LC‐MS/MS) abundance of a specified protein marker set revealed a high comparability between marker enzyme activities and marker protein abundance. Subcellular diurnal dynamics of the proteome and metabolome were recorded in leaf tissue of the hexokinase 1 deficient mutant *gin2‐1* and its wild type Landsberg *erecta* (L*er*) that finally indicated a role of hexokinase 1 in photorespiratory metabolism.

## Results

### Marker enzyme activities and LC‐MS/MS determined protein marker set are applicable for subcellular metabolome analysis

**Figure 1:**
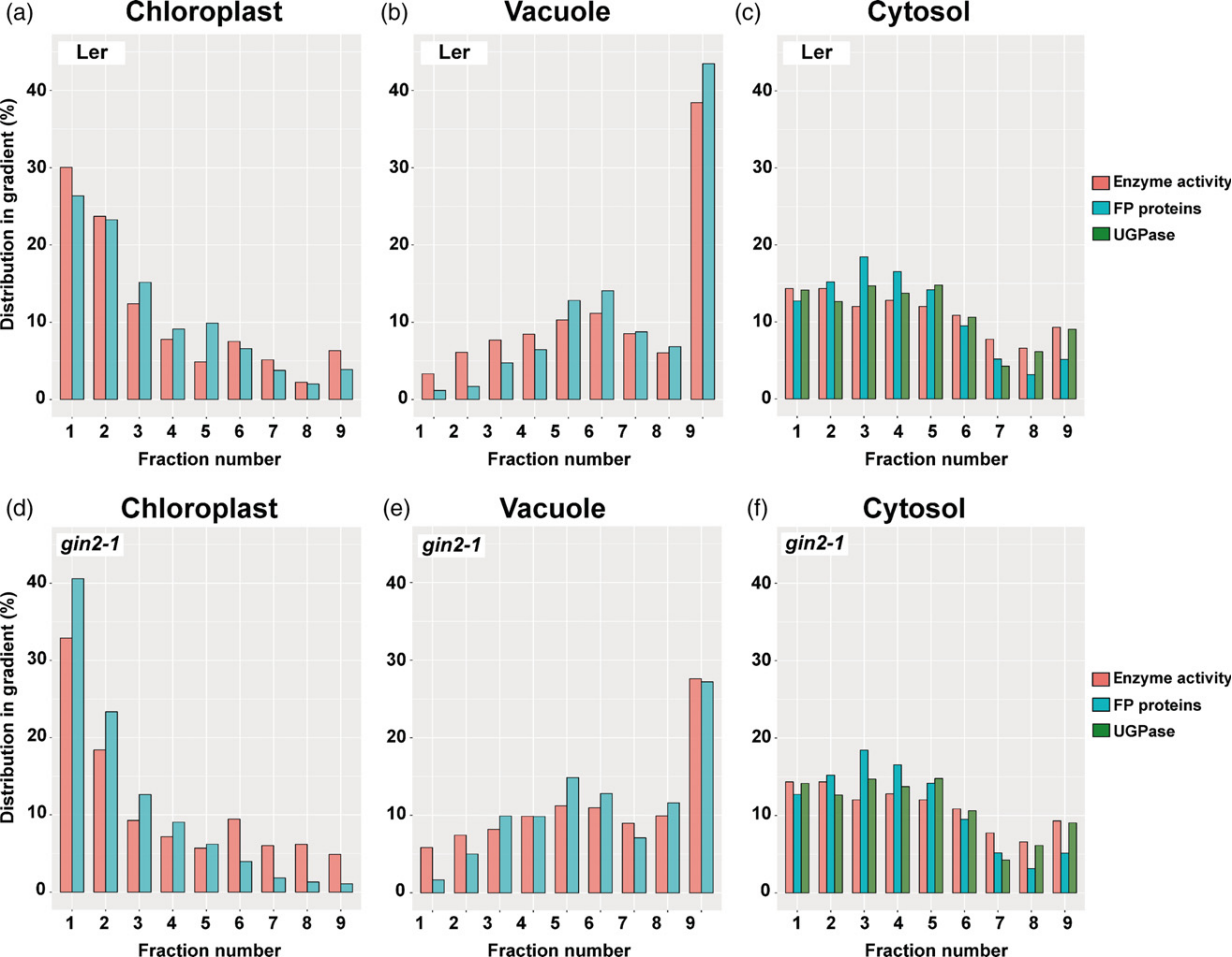
Comparison of different marker distributions in a typical gradient. (a–c) L*er* and (d−f) *gin2‐1* in three compartments (a, d: chloroplast; b, e: vacuole; c, f: Cytosol). Red colour indicates measured enzyme activities that were photometrically determined. Marker enzymes were pyrophosphatase (chloroplast), acid phosphates (vacuole) and UDP glucose pyrophosphorylase (UGPase; cytosol). Cyan bars represent mean values of all subcellular marker proteins (LC‐MS/MS; Table S1). For cytosol, protein abundance is additionally provided for cytosolic UGPase (green bars) to allow the direct comparison to enzyme activity (red bars).

Calculation of subcellular metabolite distribution critically depends on data on compartment‐specific marker enzyme activities. Typically, a single marker enzyme activity is determined for each subcellular compartment and, subsequently, correlated with metabolite levels across all fractions. To evaluate the robustness of the metabolite correlation output, calculations based on marker enzyme activity data were compared with calculations based on a diverse set of LC‐MS/MS quantified marker proteins. Marker enzyme activities were quantified for chloroplast (pyrophosphatase), vacuole (acid phosphatase) and cytosol (UGPase; see Figure 1, red bars). Additionally, protein abundance was quantified for cytosolic UGPase (AT3G03250; Figure 1c/f, green bars). Mean enzyme activity and protein abundance of UGPase differed less than 4% for both accessions across all time points and fractions (L*er*: 3.6% and *gin2‐1*: 3.4%). From a shotgun proteomic dataset across gradient fractions, proteins were considered as a subcellular marker if they were previously confirmed by fluorescent protein fusion (FP) to be solely present in one compartment (Table S1). This marker protein set comprised 11 cytosolic, two mitochondrial, four nuclear, 47 plastidial, four vacuolar and two peroxisomal proteins.

Information on FP‐proteins and their localizations were derived from SUBA3 (Tanz *et al*., 2012) and the annotator database The Arabidopsis Information Resource (TAIR) (see Experimental procedures, Table S1). FP‐proteins with unique subcellular localization were used as protein marker set within gradients (Figure 1, turquoise bars). Comparison of measured enzyme activities and the FP‐protein marker set (Figure 1) revealed high Pearson correlation coefficients in plastids (0.97) and vacuoles (0.94), while it was slightly lower for the cytosol (0.73). Lowered cytosolic correlation coefficients resulted from eight out of 163 cytosolic marker proteins that showed a deviation of ≥10% to marker enzyme activity (UGPase). As a consequence, a threshold of ≥10% for marker slope differences was used to determine subcellular metabolite distributions by the calculation algorithm to avoid overestimation due to technical limitations of marker quantification (see Experimental procedures). This 10% threshold slightly reduced the separation of subcellular metabolite clusters (see Figure S2) but increased its robustness against outliers. Besides the above described outliers, overall relative distribution of cytosolic enzyme activity and protein marker sets differed only slightly (<5%). An example for the heterogeneity of FP marker within gradients is provided in Figure S3. This low deviation indicated that quantification of single marker enzyme activities provides robust information about compartmental enrichment. Alternatively, these findings provide evidence for the suitability of shotgun proteomics to resolve subcellular metabolism.

### Protein marker correlation reveals the limitation of subcellular resolution

**Figure 2:**
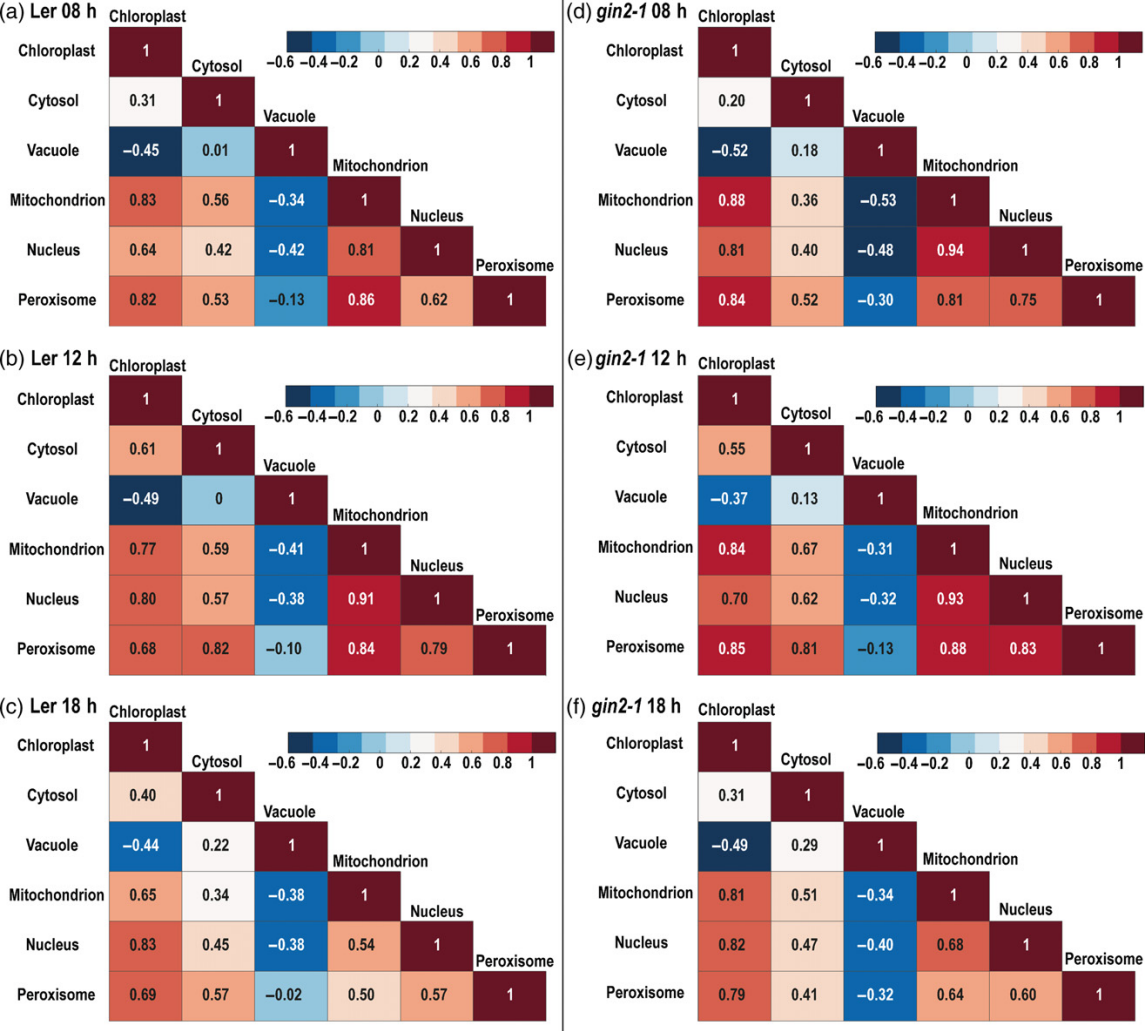
Heatmap of mean Pearson correlation coefficients of subcellular marker proteins. For each time point, compartment protein markers of each fractionated sample were correlated with each other. Results are provided as mean values of three biological replicates (*n* = 3) for (a–c) L*er* and (d–f) *gin2‐1*.

**Figure 3:**
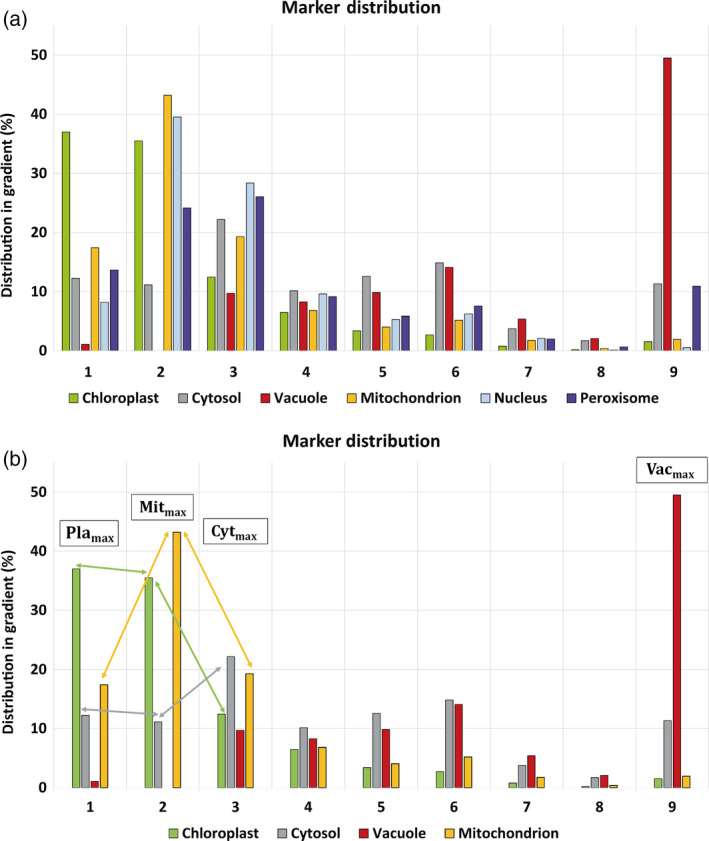
LC‐MS/MS subcellular marker distribution for a typical gradient. (a) Mean marker distribution across a nonaqueous density gradient. Mean values were built for all marker proteins listed in Table S1. (b) Compartment marker dynamics applied for correlation with metabolites. Arrows exemplarily indicate dynamics of plastidial, mitochondrial and cytosolic marker proteins which were compared with dynamics of metabolites in the same gradient. Maxima (‘max’) of compartment markers are exemplarily depicted. Comparison of fractions 1 and 2 indicates positive slopes for mitochondrial markers, and slightly negative slopes for chloroplasts and cytosol (both less than 10% and therefore not considered for metabolite distributions). Comparison of fractions 2 and 3 revealed negative slopes for chloroplasts and mitochondria and a positive slope for cytosol. Chloroplast (Pla, green), cytosol (Cyt, grey), vacuole (Vac, red), mitochondria (Mit, yellow), nucleus (pale blue), peroxisome (purple).

Subcellular protein marker sets from NAF gradients were correlated against each other to statistically evaluate potential overlaps of compartment markers. Mean values of Pearson correlation coefficients were determined for both genotypes and time points (Figure 2). Lower correlation coefficients in L*er* indicated a superior separation efficiency compared with *gin2‐1*. Time point comparison revealed the best separation of compartments at 18:00 h for both genotypes. In general, from all resolved compartments vacuole and cytosol were separated best with negative or low correlation coefficients to the other compartments. Compartments like chloroplasts, mitochondria, nucleus and, to lesser extent, also peroxisomes showed their relative maxima within the first five gradient fractions while they were low abundant in the last four fractions (Figure 3a). Mitochondria and chloroplasts showed coefficients lower than 0.85 except for *gin2‐1* at 08:00 h (*r* = 0.88). Nuclei clustered together with mitochondria as indicated by correlation coefficients >0.9 (Figure 2b,d,e). Except for 18:00 h, nucleus and peroxisomes were associated more to mitochondria than to chloroplasts. An overview of full proteome correlation against compartment marker proteins is summarized in Table S2 indicating which proteins were positively correlated with compartment marker proteins (Table S1).

### Resolving mitochondrial metabolism

**Figure 4:**
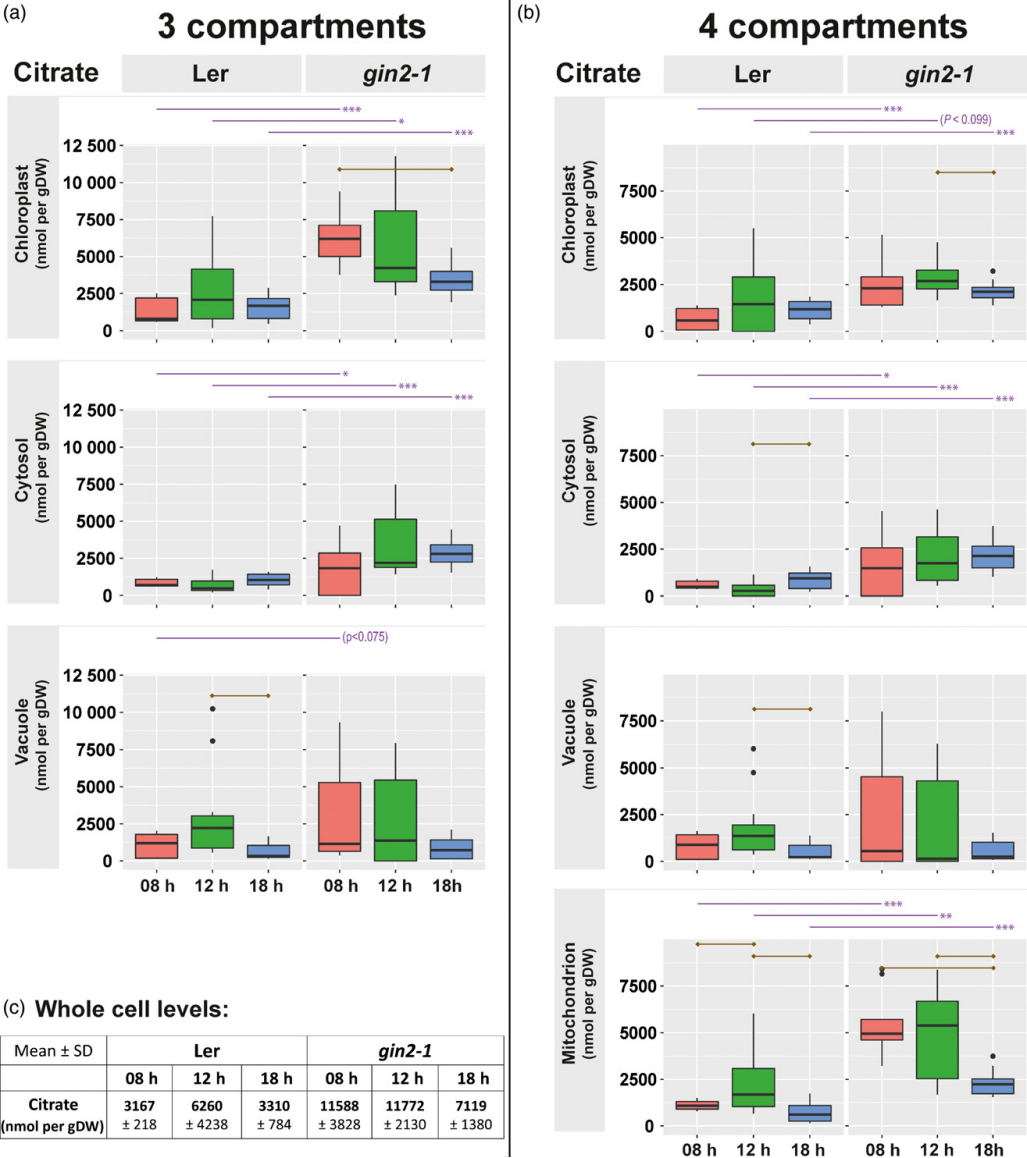
Subcellular citrate dynamics. Citrate amount was resolved for (a) three compartments (chloroplast, cytosol, vacuole) and (b) four compartments (+ mitochondria). (c) Absolute amount of citrate on the whole cell level (mean ± SD,* n* = 5). Amount is given in [nmol/gDW]. Dry weight refers to the total weight of the sample. Purple lines above graphs indicate a significant difference between genotypes at the same time point (*n* = 3 × 5; *t*‐test, *P < 0.05, **P < 0.01, ***P < 0.001). Brown lines within graphs indicate a significant difference between time points (*t*‐test, *P* < 0.05, Bonferroni correction of multiple comparisons).

Comparing the gradient distribution of plastidial, mitochondrial and cytosolic FP marker proteins indicated the location of mitochondria between plastids and cytosol (Figure 3a). The reliability of correlation output of marker proteins and metabolites critically depends on differential dynamics of compartment markers across density gradients (Figure 3b). Consequently, mathematical assignment of metabolites strongly depends on the number of compartments that are considered for each calculation. As a result, several significant correlations of metabolites with a three‐compartment model, comprising chloroplast, cytosol, and vacuole, changed their compartmental association, when a four‐compartment model was applied for correlation, for example by adding mitochondria. Citrate shifted its association towards mitochondria (Figure 4). Citrate is an intermediate of the tricarboxylic acid (TCA) cycle and, thus, this indicated that the four‐compartment model improved the estimation of subcellular metabolite localization. In total, the mitochondrial four‐compartment model significantly affected about 33% of quantified metabolites of the three‐compartment model in *gin2‐1* while only 19% were affected in L*er* (Table S3, *t*‐test *P* < 0.05). This might be the result of a better separation of compartments in L*er* that became visible in the Pearson correlation coefficients (Figure 2).

### A mitochondrial four‐compartment model

The impact of the mitochondrial four‐compartment model is exemplarily shown for the TCA cycle intermediate citrate by comparing a three‐compartment calculation (Figure 4a) with a four‐compartment calculation (Figure 4b). Involving mitochondrial markers in subcellular correlation cancelled out a diurnal decrease of citrate within the chloroplast that was observable for *gin2‐1* in the three‐compartment model (Figure 4a,b). Generally, mean values of citrate in mitochondria were highest compared with the other three compartments for both genotypes across all three time points, except for L*er* at 18:00 h (Figure 4b). In *gin2‐1*, significantly higher citrate levels than in L*er* were observed in mitochondria, cytosol and chloroplasts (Figure 4b, *t*‐test, *P* < 0.05). Within mitochondria, lowest citrate levels were observed at 18:00 h in both genotypes.

### *gin2‐1* is significantly affected in hexose allocation between cytosol and vacuole

**Figure 5:**
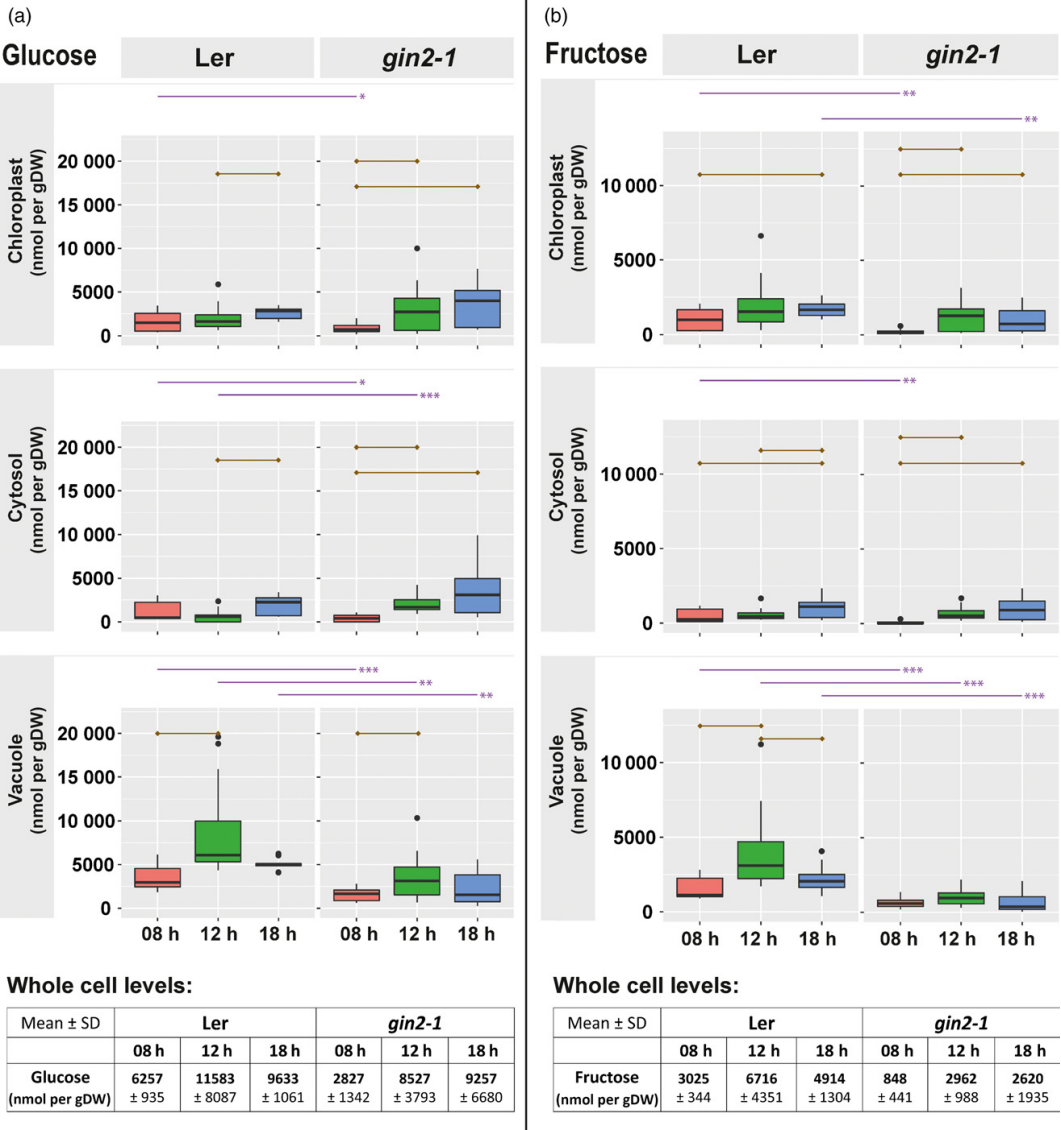
Hexose amount in three compartments. (a) Glucose and (b) fructose amount in [nmol/gDW] for chloroplast, cytosol and vacuole. Dry weight refers to the total weight of the sample. Purple lines above graphs indicate a significant difference between genotypes at the same time point (*n* = 3 × 5; *t*‐test, *P < 0.05, **P < 0.01, ***P < 0.001). Brown lines inside of graphs indicate a significant difference between time points (*t*‐test, *P* < 0.05, Bonferroni correction of multiple comparisons). Tables below graphs contain absolute amount of metabolites at a whole cell level (mean ± SD,* n* = 5).

Total amounts of glucose and fructose were significantly lower in *gin2‐1* than in L*er* at the beginning of the day. In L*er*, hexose amount increased significantly until 18:00 h (table in Figure 5; *P* < 0.05). Hexose amount in *gin2‐1* was significantly lower than in L*er* across all three compartments at 08:00 h (Figure 5; *t*‐test *P* < 0.05). Estimated mitochondrial hexose dynamics indicated a linear increase of glucose amount in L*er*, while in *gin2‐1* a significant increase of hexoses was only found during the first 4 h in the light (Figure S7; *t*‐test *P* < 0.05). Cytosolic and plastidial hexose levels of *gin2‐1* were less affected than vacuolar levels that were significantly reduced to ~50% of L*er* (*P* < 0.01). *gin2‐1* continuously accumulated glucose in the cytosol, while L*er* showed lowered levels at 12:00 h.

### Pyruvate and TCA cycle intermediates are significantly affected in mitochondria of *gin2‐1*

**Figure 6:**
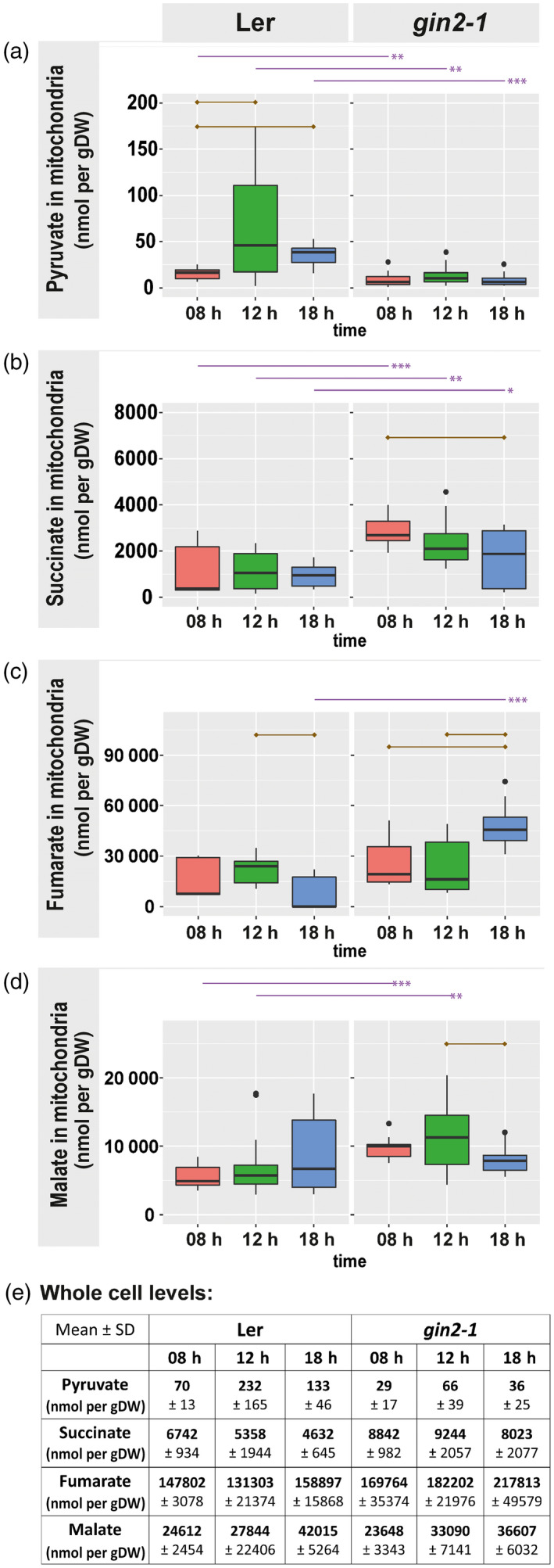
Pyruvate and TCA cycle intermediates in mitochondria. (a) Pyruvate, (b) succinate, (c) fumarate and (d) malate in [nmol/gDW] for both genotypes. (e) The table contains absolute amounts of pyruvate, succinate, fumarate and malate at a whole cell level (mean ± SD,* n* = 5). Dry weight refers to the total weight of the sample. Purple lines above graphs indicate significant differences between genotypes at the same time point (*n* = 3 × 5; *t*‐test, *P < 0.05, **P < 0.01, ***P < 0.001). Brown lines inside of graphs indicate significant differences within genotypes (*t*‐test, *P* < 0.05, Bonferroni correction of multiple comparisons).

In *gin2‐1*, mitochondrial pyruvate values were significantly lower than in L*er* across all time points (Figure 6a; *P* < 0.01). Furthermore, in L*er*, pyruvate levels increased over the day whereas no significant dynamics were observed in *gin2‐1*. Similar to citrate (Figure 4b), mitochondrial succinate levels were significantly higher in *gin2‐1* than in L*er* across all time points. Additionally, the dynamics of mitochondrial succinate differed between both genotypes, showing a significant decrease in *gin2‐1* and constant levels in L*er* (Figure 6b). Estimated mitochondrial fumarate amount reached a maximum in *gin2‐1* at 18:00 h (Figure 6c). Mitochondrial malate amount was significantly higher in *gin2‐1* than in L*er* at 08:00 and 12:00 h (Figure 6d). A summary of metabolite levels across all compartments is provided in Figure S4.

### Photorespiration associated amino acids glycine and serine are less abundant in g*in2‐1*

**Figure 7:**
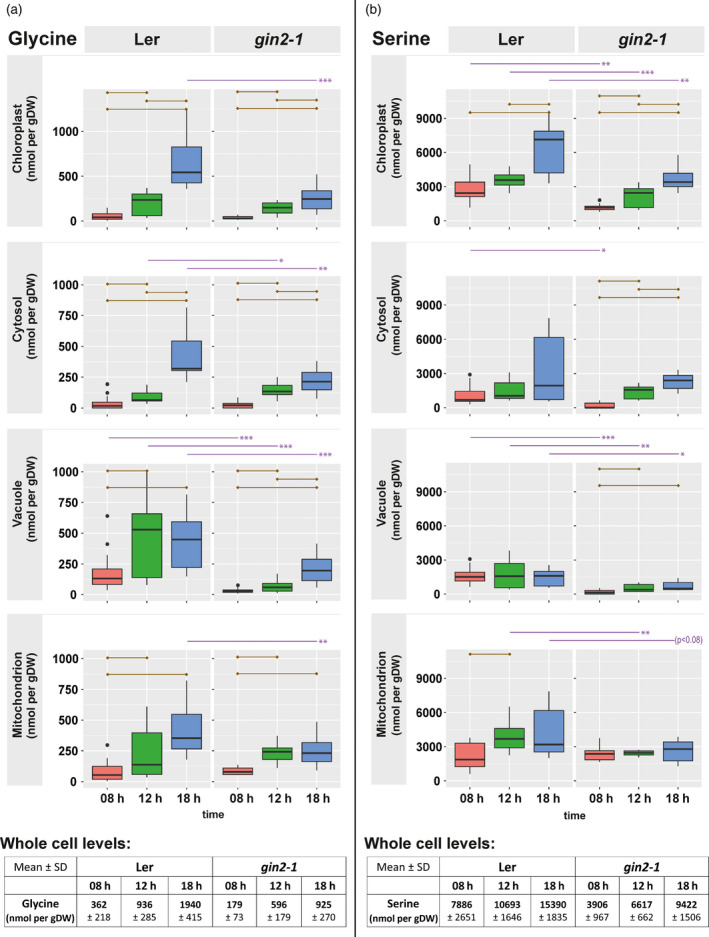
Subcellular amino acid amount. (a) Glycine and (b) serine in [nmol/gDW] for both genotypes. Dry weight refers to the total weight of the sample. Purple lines above graphs indicate significant differences between genotypes at the same time point (*n* = 3 × 5; *t*‐test, *P < 0.05, **P < 0.01, ***P < 0.001). Brown lines inside of graphs indicate significant differences within genotypes (*t*‐test, *P* < 0.05, Bonferroni correction of multiple comparisons). The tables below the graphs contain absolute metabolite amounts at a whole cell level (mean ± SD,* n* = 5, Bonferroni correction of multiple comparisons).

Glycine accumulated across all compartments and genotypes, but at 18:00 h the amount was significantly lower in *gin2‐1* compared with L*er* (Figure 7a; *P* < 0.01). In more detail, mitochondrial glycine levels in L*er* increased over the whole light period, whereas in *gin2‐1* glycine levels remained constant between 12 and 18:00 h. Similarly, serine accumulated in chloroplasts and in the cytosol of both genotypes (Figure 7b; *P* < 0.05), while plastidial levels were significantly lower in *gin2‐1*. In L*er*, mitochondrial serine levels significantly increased during the light phase (Figure 7b, *P* < 0.05) but remained constant and significantly lower (*P* < 0.05) in *gin2‐1*. Vacuolar serine levels were found to be significantly lower in *gin2‐1* than in L*er* (Figure 7b, *P* < 0.05). Additionally, hexokinase 1 deficiency affected amino acid levels at the C/N interface (glutamine, glutamate, Figure S5a,b) and to a strong depletion of proline (Figure S5c). All other quantified amino acids showed significant differences between genotypes and time points in at least one compartment (summarized in Tables S4 and S5).

### Subcellular proteomics reveals a HXK1‐induced effect on diurnal regulation of mitochondrial metabolism

**Figure 8:**
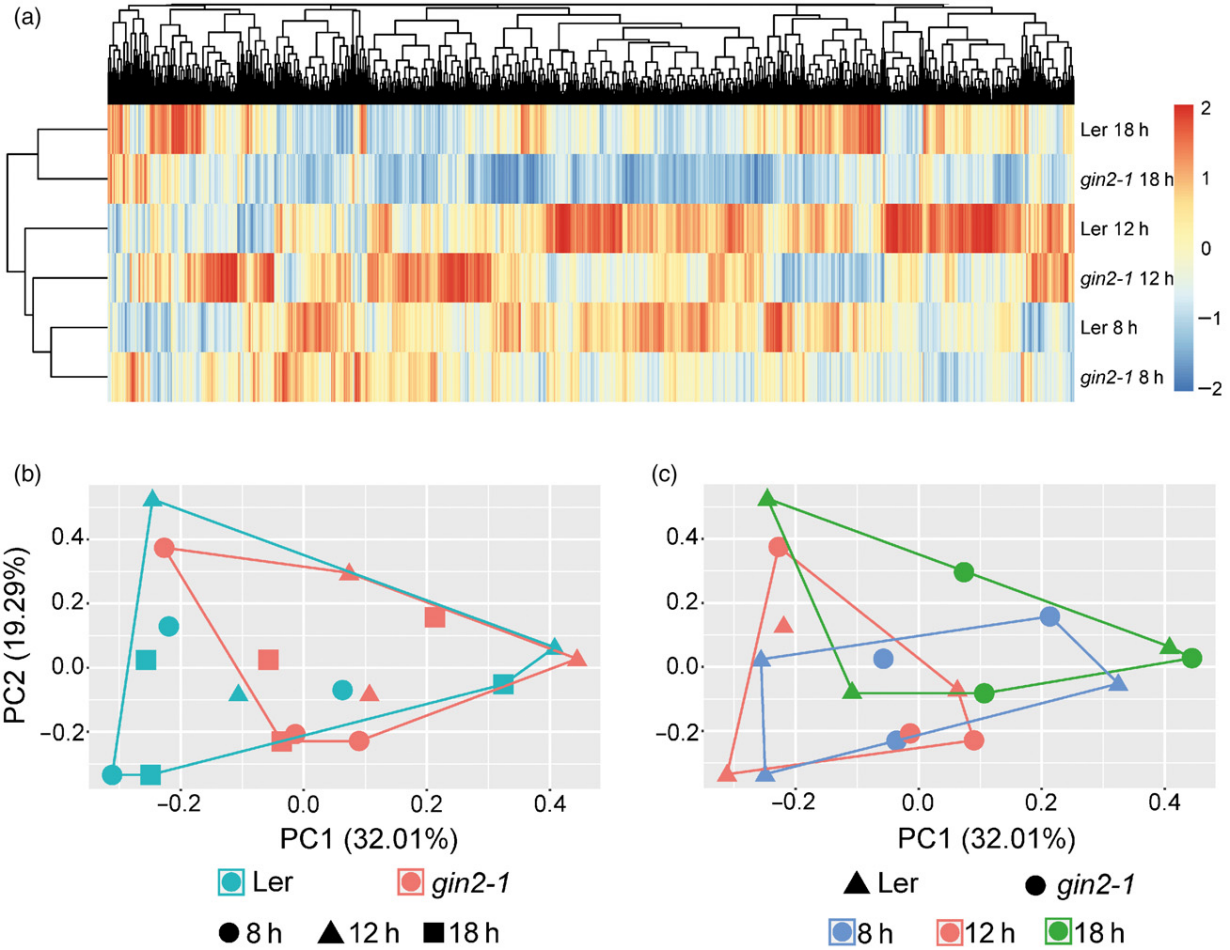
Multivariate analysis of diurnal proteome dynamics in L*er* and *gin2‐1*. (a) Hierarchical clustering of proteome dynamics based on Euclidean distance of mean values for genotype and time point (*n* = 3). Colour bar indicates scaled protein abundance across all genotypes and time points. Proteome dynamics were analyzed by principal component analysis summarizing (b) genotype effects, and (c) diurnal dynamics. A significant genotype effect was observed for 145 proteins, while the diurnal effect is comprised of 60 significantly changed proteins in L*er* and 105 significant diurnal changes in gin2‐1 (ANOVA,* P* < 0.05, Bonferroni correction of multiple comparisons).

**Figure 9:**
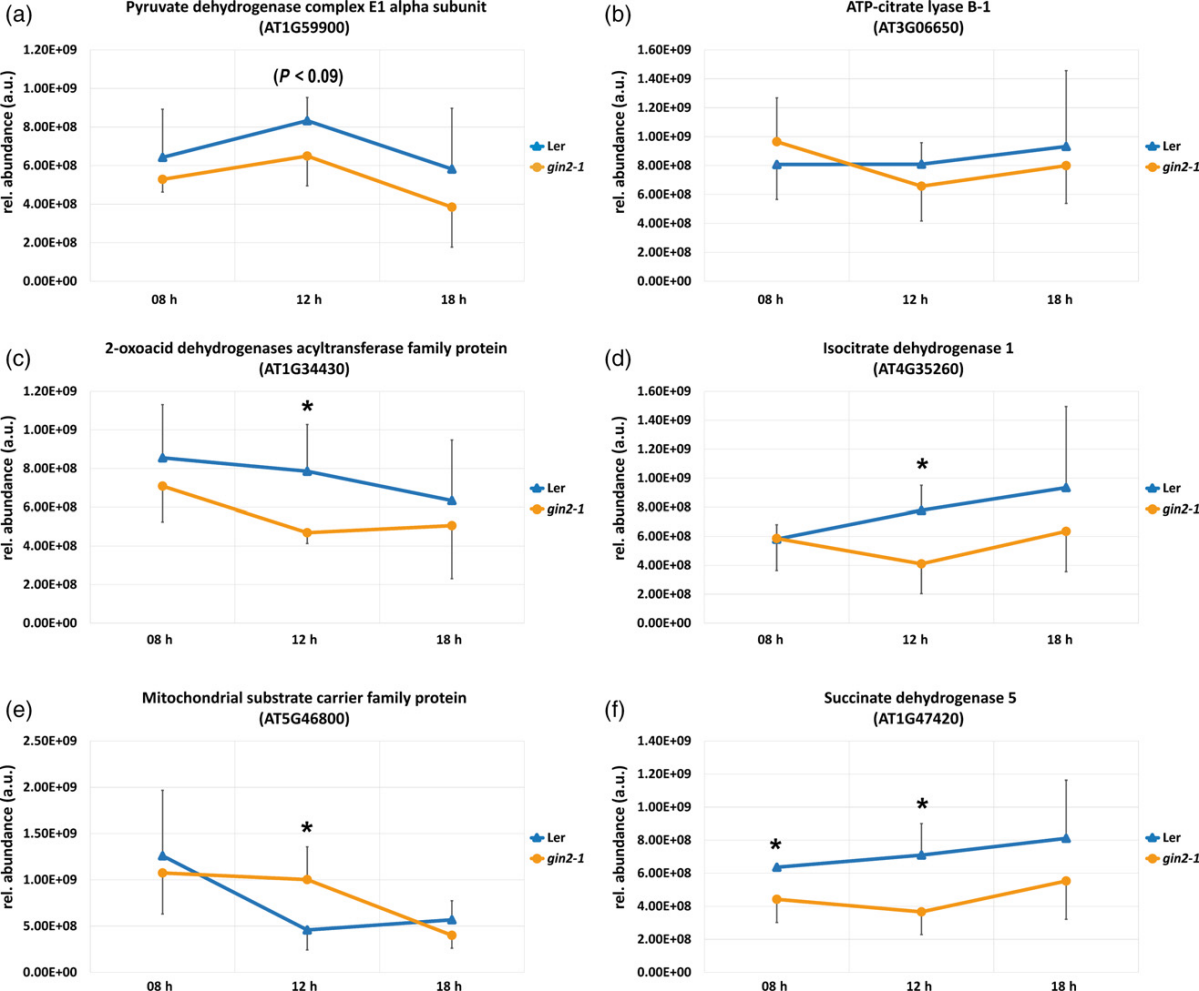
Proteins involved in the TCA cycle and organic acid transformation reactions discriminate L*er* and *gin2‐1*. Candidates were selected by Venn analysis of the high abundance quartile at 12 h. (a–d) Diurnal dynamics of proteins derived from the high abundance quartile in L*er*. (e) Diurnal dynamics of the putative mitochondrial transporter Bou. (f) Succinate dehydrogenase 5 (mean ± SD,* n* = 3). Asterisks indicate significant differences between L*er* and *gin2‐1* (*t*‐test; *P < 0.05).

Diurnal dynamics of protein levels indicated a similar cellular protein constitution in L*er* and *gin2‐1* during the early morning and evening phase, while differences in mean proteome abundance between both genotypes peaked after 4 h into the light (Figure 8a). However, principal component analysis showed that neither genotype nor time points were separated by the proteomics data (Figure 8b,c). Comparing the quartile of most abundant proteins, i.e. 750 out of 2995 with highest abundance in L*er* and *gin2‐1* at 12:00 h, revealed an overlap of 681 proteins while 69 proteins were more abundant in either L*er* or *gin2‐1* (Figure S6). Functional categories (TAIR10 (www.arabidopsis.org) protein database (Lamesch *et al*., 2012)) of the most abundant proteins revealed that a particular set of proteins, involved in TCA cycle and organic acid transformation reactions, was underrepresented in *gin2‐1* (Figure 9). These proteins comprised the pyruvate dehydrogenase complex E1 alpha subunit (E1 alpha; AT1G59900, Figure 9a), ATP‐citrate lyase B‐1 (AT3G06650, Figure 9b), 2‐oxoacid dehydrogenases acyltransferase family protein (AT1G34430, Figure 9c) and isocitrate dehydrogenase 1 (AT4G35260, Figure 9d). At 12:00 h, levels of 2‐oxoacid dehydrogenases acyltransferase family protein and isocitrate dehydrogenase 1 were significantly higher in L*er* than in *gin2‐1* (*P* < 0.05; *t*‐test), while levels of E1 alpha and ATP‐citrate lyase B‐1 were only slightly elevated (Figure 9a–d; *P* = 0.09 and *P* = 0.2). Alternatively, the abundance of the putative mitochondrial transporter Bou (AT5G46800, Figure 9e) was higher in *gin2‐1* after 4 h in the light, because of a decrease during the morning phase in L*er* (*P* = 0.07; Figure 9e). In *gin2‐1*, Bou levels decreased significantly 6 h later (*P* < 0.05, *t*‐test).

**Figure 10:**
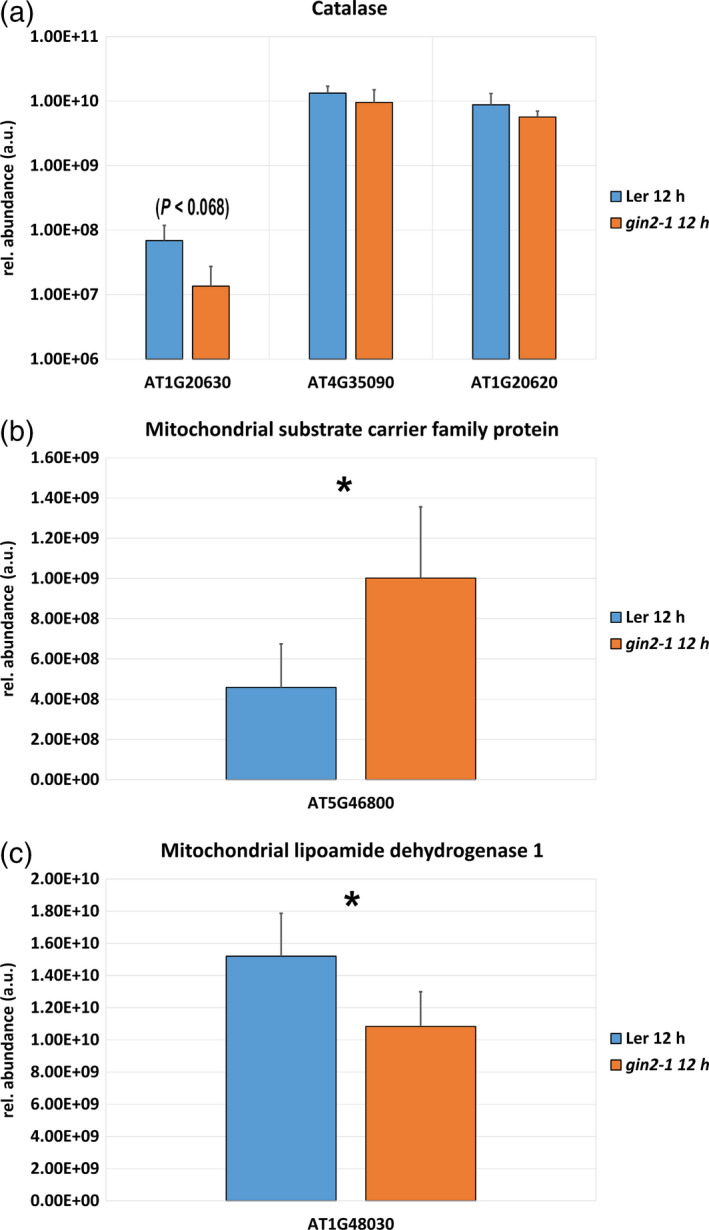
Mitochondrial protein levels in L*er* and *gin2‐1* under ambient growth conditions at 12 h. (a) Relative abundance of catalase isoform Cat1 (AT1G20630), Cat2 (AT4G35090) and Cat3 (AT1G20620). (b) Relative abundance of the putative mitochondrial transporter A BOUT DE SOUFFLE, Bou (AT5G46800). (c) Relative abundance of mitochondrial lipoamide dehydrogenase 1 (mLPD1, AT1G48030) (mean ± SD,* n* = 3). Asterisks indicate significant differences (*t*‐test; *P < 0.05).

These observations indicated a HXK1‐dependent diurnal regulation of mitochondrial metabolism. For a more detailed analysis at the subcellular level, the full proteome was correlated with marker proteins for the compartments plastid, cytosol, vacuole, mitochondrion and nucleus (significant Pearson correlation, see Experimental procedures). In total, 2786 out of 2995 proteins (93%) significantly correlated with at least one compartment in either L*er* or *gin2‐1* (Pearson correlation, *P* < 0.05). The overlap of significant correlations between both genotypes was ~75%. To reveal the effect of the *gin2‐1* mutation on mitochondrial metabolism, proteins showing strongest significant correlation with mitochondrial markers in L*er* were extracted from the data set and compared with *gin2‐1*. In total, 383 proteins in L*er* and 586 proteins in *gin2‐1* were found to significantly correlate with mitochondrial markers over the complete diurnal period, and 94 of these proteins were only found in L*er*. Among these 94 proteins, catalase 1 (Cat1; AT1G20630) showed the weakest association with any other compartment, for example with the nucleus, indicating a strong and exclusive association with mitochondria. At 12 h, Cat1 levels of L*er* were five‐fold higher than in *gin2‐1* (Figure 10a; *P* = 0.068). Less significant, catalase isoforms Cat2 and Cat3 were slightly more abundant in L*er* than in *gin2‐1* (Figure 10a). In *gin2‐1*, strongest association with mitochondria over the diurnal period was observed for the putative mitochondrial transporter A BOUT DE SOUFFLE (BOU; AT5G46800), NADH dehydrogenase [ubiquinone] 1 alpha subcomplex subunit 6 (AT3G12260) and NADH dehydrogenase [ubiquinone] 1 beta subcomplex subunit 7 (AT2G02050). At 12 h, Bou protein levels were 2‐fold higher in *gin2‐1* than in L*er* (Figure 10b; *P* < 0.05). In a previous study, Bou was identified as a transporter involved in photorespiratory metabolism by co‐expression analysis using 14 highly co‐expressed photorespiratory genes (Eisenhut *et al*., 2013), and seven of the encoded proteins were also quantified in the present study. Those proteins comprised serine hydroxymethyl transferase 1 (SHM1; At4g37930), glycine decarboxylase (GDC) L‐protein (mLPD1; At1g48030), glyocolate oxidase (GOX; At3g14415), hydroxypyruvate reductase 1 (HPR1; At1g68010), glutamate:glyoxylate aminotransferase 2 (GGT2; At1g23310), glutamine synthetase 2 (GS2; At5g35630) and the Fd‐dependent glutamate synthase (fd‐GOGAT; At5g04140). Among these seven proteins, mLPD1 levels at 12:00 h showed the most significant difference between both genotypes, being 1.4‐fold higher in L*er* than in *gin2‐1* (Figure 10c; *P* < 0.05). Although not always significant, levels of many detected photorespiratory proteins were higher in L*er* than in *gin2‐1* over the whole diurnal period.

## Discussion

### Subcellular resolution of metabolism

Resolving the dynamics of subcellular metabolite concentrations is essential to promote the understanding of cellular biochemistry and metabolic regulation. The NAF technique combines efficient quenching of metabolism with subcellular resolution by correlation of marker proteins or enzymes with metabolites. Although genetically encoded or optical sensors for metabolites enable spatiotemporal tracing of subcellular dynamics in single metabolite pools (Bermejo *et al*., 2011; De Col *et al*., 2017; Depaoli *et al*., 2018; Zhang *et al*., 2018), NAF is still the method of choice to comprehensively resolve metabolism from one sample. Previously, NAF was combined with a proteomics and metabolomics approach to increase the coverage of metabolic analysis (Arrivault *et al*., 2014). These authors proved the NAF method to be suitable for separation and quantification of subcellular proteins and metabolites, but also indicated the difficulty in separating cytosol from mitochondria and peroxisomes. Similarly, the present study shows that marker protein distribution across a NAF density gradient matches the distribution of marker enzyme activities for the compartments plastid, cytosol and vacuole. The combined metabolite‐protein analysis was applied to resolve diurnal dynamics of subcellular metabolism in *gin2‐1* and L*er*. Here, 47 plastidial protein markers (Table S1) were used and compared with plastidial pyrophosphatase enzyme activities, which matched a high Pearson correlation coefficient (>0.97) for both genotypes (Figure 1a,d). This also held true for the vacuole, where acid phosphatase enzyme activities and abundance of four determined vacuolar proteins matched with a correlation coefficient of >0.94 (Figure 1b,e). UGPase activity and UGPase protein abundance matched with mean discrepancies of 4%, and similar results were obtained when FP marker proteins were compared (Figure 1c,f).

Based on marker distribution across a density gradient, subcellular metabolite distributions are calculated applying correlation strategies that approximates the *in vivo* concentrations in subcellular compartments. Therefore, choice of correlation strategies is critical for calculation of subcellular metabolite concentrations (Dietz, 2017). Here, only those gradient fractions that differed by at least 10% in marker protein content were used for metabolite correlation. With this threshold, over‐interpretation of weak and insignificant marker dynamics across nonaqueous density gradients was prevented.

In contrast with subcellular metabolomics, NAF results for subcellular proteomics can be cross‐validated using methods such as immunofluorescence. Additionally, based on amino acid sequences, subcellular localization of proteins can be predicted using programs such as ChloroP (Emanuelsson *et al*., 1999), MITOPROT (Claros and Vincens, 1996), or TargetP (Emanuelsson *et al*., 2007). Analysis and correlation of the shotgun resolved proteome with compartment‐specific FP marker proteins revealed a high consensus with correlations based on marker enzyme activities (Figure 1) that had also been observed before (Arrivault *et al*., 2014). Therefore, although numbers of marker proteins for each compartment differed considerably (see Table S1), a robust correlation output was observed that clearly indicated the suitability of both marker enzyme activities and marker proteins for subcellular correlation. This finding provides evidence for the suitability of NAF to cross‐validate and supplement predictions on subcellular protein localization. Besides that, correlation with validated marker proteins of other compartments revealed the subcellular localization of, hitherto, experimentally uncharacterized proteins (Table S2). This application, however, is limited by the overlap of marker proteins of two or more compartments. Previously, mitochondria were found to cluster together with the cytosol, and this clearly limited the resolution of cellular primary and energy metabolism (Arrivault *et al*., 2014). Here, no unambiguous explanation can be given why mitochondria and cytosol could be separated in the present study. However, both studies differed in aspects of plant material, growth conditions and applied nonaqueous gradients. While Arrivault and colleagues analyzed *Arabidopsis thaliana*, accession Col‐0, the present study used the accession L*er*, which is the genetic background of *gin2‐1* mutants. Although it is highly speculative at this point, different number, size and ultrastructure of mitochondria in both genotypes might explain the observed differences. Second, while in the present study plants were grown under long day conditions (16/8 h) and with PAR 120 μmol m^−2^ sec^−1^, growth conditions applied by Arrivault and colleagues were 12/12 h and 150 μmol m^−2^ sec^−1^. Although light intensity differed only by 20%, mitochondrial shape and ultrastructure has previously been described to depend on light and cytosolic sugar concentrations and this might contribute to the observed differences (Jaipargas *et al*., 2015). Additionally, quality of compartment separation was found to vary over diurnal cycles and genotypes (see Figure 2). For example, best separation of mitochondria and plastids was observed during the late afternoon and was better in L*er* than in *gin2-1* (Figure 2c,f). To further validate the separation of mitochondrial from cytosolic and plastidial metabolism, in future studies NAF might be applied to sensor lines, for example ATP sensor lines (De Col *et al*., 2017) that would cross‐validate both methodologies. Our data indicated that both diurnal sampling time point and genotype affect the quality of NAFs, and, therefore, automatically affect the reliability of estimated subcellular metabolite distribution. Finally, in the present study, non‐aqueous density gradients comprised nine fractions, while Arrivault and colleagues applied a 12‐fraction gradient. In general, a high number of subfractions provides robust correlation output and, potentially, can resolve more subcellular compartments. Yet, it also dilutes concentrations of marker proteins and enzymes across the subfractions. Choosing the number of density gradient fractions remains a compromise between the signal‐to‐noise ratio of protein detection and the number of compartments that are aimed to be resolved.

### Assignment of metabolites to multiple compartments

Increasing the resolution of NAF to more than three compartments led to displacements of relative and absolute values in some or all compartments, therefore affecting significance of correlations of metabolites with compartments. Arrivault and colleagues reported that in their NAF experiments mitochondria matched predominantly with the cytosol (Arrivault *et al*., 2014). However, in the present study, mitochondrial marker proteins peaked either between maxima of plastidial and cytosolic markers (see Figure 3b), or overlapped with plastidial markers. Also, Pearson correlation indicated clustering of mitochondria with chloroplasts rather than with the cytosol (see Figure 2). Significant shifts towards mitochondria, obtained for 64% of resolved primary metabolites affected all three compartments. For example, including mitochondrial markers revealed the highest amount of citrate in this compartment for both genotypes at all time points (Figure 4b), except for L*er* at 18:00 h. Comparing three‐compartment with four‐compartment citrate distribution (Figure 4), no decrease in chloroplasts of *gin2‐1* from 08 to 18:00 h was observable anymore, and citrate dynamics turned out similar to that of L*er* (Figure 4b). In contrast, estimated cytosolic and vacuolar citrate amount was only slightly affected by involving mitochondrial markers, providing evidence for the suitability of this method to differentiate mitochondrial from estimated vacuolar amount of organic acids. However, a critical limitation of the suggested methodology is indicated by the subcellularly resolved sucrose metabolism, which displayed a significant shift of sucrose from plastids to mitochondria when comparing the 3‐compartment and 4‐compartment model (Tables [Link], [Link], [Link]). Therefore, while resolving mitochondrial metabolism prevents overestimation of plastidial proportions of organic acids, plastid localized metabolites, for example sugars, might be underestimated by this approach. A similar effect might explain mitochondrial hexose levels in a 4‐compartment model (see Figure S7). Together with the observed effects of genotype and time point on separation quality of the 4‐compartment model, our study indicates that NAF results have to be critically discussed in the context of further experimental evidence, for example derived from studies on sensor lines (De Col *et al*., 2017). In general, we suggest combining results from a three‐compartment and four‐compartment model and to discuss the probability of subcellular metabolite localization in context of metabolic pathways. This, however, clearly limits the applicability of NAF to targeted metabolomics approaches by which metabolites and pathways are analyzed which have been studied by other methods before. Finally, it remains to be elucidated if compartment separation might be improved by application of non‐linear density gradients applying methods like the benchtop NAF (Fürtauer *et al*., 2016).

### HXK1 deficiency affects intercompartmental hexose dynamics

Hexokinases (HXKs) constitute a gateway into glycolysis for hexoses arising from sucrose or transitory starch degradation. HXKs are abundant in most plant cell types and play a central role because they are the only known enzymes in plants which can phosphorylate glucose (Granot *et al*., 2014). Although HXK1 is known as a conserved glucose sensor that integrates environmental signals to control growth and development (Moore *et al*., 2003), its regulatory function in subcellular carbohydrate metabolism is less clear. Whole cell hexose amount showed reduced levels at the beginning of the day (08:00 h; Figure 5). Estimated cytosolic and chloroplastidial glucose amount continuously increased in *gin2‐1* (08:00–18:00 h), whereas vacuolar hexose amount were significantly higher in L*er* than in *gin2‐1* (Figure 5a,b, *P* < 0.01). This suggests an important role for HXK1 in balancing glucose concentrations across compartments. While the most significant genotype effect was observed for estimated vacuolar glucose amounts (see Figure 5a), the cytosol probably acts as a regulatory hub for subcellular glucose allocation that might dampen cytosolic glucose dynamics. Although the difference in cytosolic glucose amounts was significant between both genotypes only at 08h and 12h, dynamics of the median glucose amounts were more pronounced in *gin2‐1* than in L*er*. Glucose is the substrate for numerous metabolic processes, for example glycolysis and mitochondrial metabolism that are directly linked to conserved signalling networks of energy metabolism. Conserved protein kinases such as Target of Rapamycin (TOR) and sucrose non‐fermenting related kinase 1 (SnRK1) play a central role in cellular signal integration (Nukarinen *et al*., 2016; Li *et al*., 2019). Glucose, together with sucrose, play an indispensable role in these signalling networks. Therefore, our data suggest a role for hexokinase 1 in stabilizing cytosolic glucose homeostasis that might be critical for plant energy metabolism.

In plant mesophyll cells of leaf tissue, a significant proportion of free hexoses is generated by invertase‐driven sucrose hydrolysis. Cytosolic and vacuolar invertases have previously been found to stabilize photosynthetic carbohydrate metabolism by catalyzing a subcellular sucrose cycle (Huber, 1989; Nägele *et al*., 2010; Weiszmann *et al*., 2018). Therefore, HXK1 might stabilize sucrose cycling by reducing the concentration of free hexoses and prevent feedback inhibition of invertases. Recently it was suggested that *gin2‐1* suffers from diminished sucrose cycling capacities that directly affect carbon allocation towards roots (Küstner *et al*., 2019). Furthermore, a cytosolic glucose accumulation and feedback inhibition of sucrose cleavage might also result in a metabolic feedback regulation of photosynthesis and/or photosynthetic gene expression by transient sugar accumulation in the cytosol (Sheen, 1994, 2014). This regulation could be due to imbalanced ADP/ATP homeostasis (Weiszmann *et al*., 2018), a changed ratio of organic bound and inorganic phosphate (Büssis *et al*., 1997) and stabilization of photosynthesis by preventing phosphate depletion of chloroplasts (Stitt and Hurry, 2002).

### Metabolic indications for reduced photorespiratory capacity in *gin2‐1*

In illuminated leaves evidence was provided for a noncyclic TCA cycle (Hanning and Heldt, 1993; Tcherkez *et al*., 2009; Gauthier *et al*., 2010). Instead of a full cycle, two weakly connected branches appear to operate in opposing directions. Previous work has indicated a central role of trehalose 6‐phosphate (Tre6P) in the coordination of carbon and nitrogen metabolism in plants (Figueroa *et al*., 2016). It was suggested that Tre6P activates flux through mitochondrial pyruvate dehydrogenase (mPDH) and citrate synthase. Although not quantified in the present study, it is interesting to speculate about the role of Tre6P within the *gin2‐1* mutant. Reduced HXK1 activity was found to result in significantly higher cytosolic glucose amounts than in the wild type (see Figure 5) and might lead to feedback inhibition of the trehalose and Tre6P pathway. This might result in an increased Tre6P amount in *gin2‐1*; this is pure speculation at this point and needs to be validated in future studies. However, it might explain the observed increased in organic acids in *gin2‐1* (see Figure S4).

Many steps of the TCA cycle can be bypassed in other compartments such as cytosol or peroxisomes (Sweetlove *et al*., 2010). This might be the reason for a lack of one specific trend for all TCA cycle intermediates observed in mitochondria of both genotypes (Figures 4b and 6). TCA cycle protein abundances were reduced in *gin2‐1* (Figure 9), while organic acids such as succinate and malate accumulated (Figures 6 and S4). Mitochondrial succinate is oxidized by succinate dehydrogenase (SDH), which also acts as a component of complex II in the electron transport chain. Phytochrome A regulates SDH expression, and this mechanism is probably important for regulation of mitochondrial respiration in the light (Popov *et al*., 2010). SDH can be a limiting factor in plant growth, as inhibition of SDH leads to downregulation of genes related to the cell cycle (Jardim‐Messeder *et al*., 2015). Therefore, lowered SDH protein levels observed in *gin2‐1* (Figure 9f) could be an additional factor limiting growth (Figure S8).

Under conditions of excessive formation of redox equivalents, photorespiration functions as a sink for electrons and ATP, therefore constituting an emergency valve in plants (Heber, 2002; Scheibe, 2004). Proteomic analysis of L*er* and *gin2‐1* at the subcellular level revealed that diurnal dynamics of proteins involved in mitochondrial metabolism and photorespiration differed between L*er* and *gin2‐1* (Figures 9 and 10). Photorespiration constituted the highest metabolic flux in mitochondria under photosynthetic conditions (Obata *et al*., 2016). A close connection between TCA cycle activity and the photorespiratory pathway exists, as alterations in TCA metabolism are observed in photorespiratory mutants and *vice versa*. The mitochondrial carrier protein Bou (AT5G46800) has previously been described to be involved in photorespiratory metabolism (Eisenhut *et al*., 2013). The analysis of a *bou* knockout‐mutant revealed a significant accumulation of glycine when compared with the wild type, and it was hypothesized that this accumulation was due to impaired glycine oxidation in the mitochondria (Eisenhut *et al*., 2013). Photorespiratory amino acids glycine and serine showed diminished levels in several compartments in *gin2‐1* (Figure 7). Although glycine accumulation was observed in both genotypes during the day, it was more pronounced in L*er* than in *gin2‐1* (Figure 7a). Additionally, serine amount increased in mitochondria of L*er*, while no diurnal dynamics were observed in *gin2‐1* (Figure 7b). Glycine is mainly provided by photorespiration, and is required for the formation of glutathione, which is an important ROS scavenger during oxidative stress (Noctor *et al*., 1999; Wingler *et al*., 2000). Higher levels of serine are known to act as a metabolic signal to induce and control transcription of photorespiration‐related genes and proteins (Timm *et al*., 2013). These findings let us speculate about a higher photorespiratory capacity in L*er* than in *gin2‐1* under ambient growth conditions, which might contribute to the discrepancy of growth rate (Figure S8) and photosynthetic CO_2_ assimilation (Brauner *et al*., 2015). Furthermore, it suggests that HXK1 is involved in the regulation of mitochondrial transport processes that affect photorespiratory metabolism.

## Experimental procedures

### Plant material

*Arabidopsis thaliana* (L.) Heynh., accession Landsberg *erecta* (L*er*) and *gin2‐1* (ABRC line N6383; At4g29130, *gin2–1* mutant) were grown in a 1:1 mixture of GS90 soil and vermiculite in a growth chamber. Light intensity was set to 120 μmol m^−2^ sec^−1^ in an 8 h/16 h day/night 22°C/16°C cycle with 70% relative air humidity. Plants were fertilized with a nitrogen−phosphate−potassium fertilizer (Wuxal^®^ Top N, Hauert MANNA Düngerwerke GmbH, Nürnberg). After 5 weeks, plants were transferred to long‐day conditions within a 16 h/8 h day/night regime. Eight days after transfer to long‐day condition plants were harvested at three time points: (i) immediately before the light was turned on (‘08:00 h’), (ii) after 4 h of light (‘12:00 h’) and (iii) after 10 h of light (‘18:00 h’). Sampling was performed under the light intensity at which plants were growing. All samples were immediately frozen in liquid nitrogen, frozen leaves were ground to a fine powder using a MM200 ball mill (Retsch GmbH) and stored at −80°C until further use. For L*er*, each biological replicate consisted of one leaf rosette while, for *gin2‐1*, two rosettes were pooled for one independent sample in the *gin2‐1* mutant to gain sufficient leaf material for NAF.

### Nonaqueous fractionation

NAF was performed as described earlier (Nägele and Heyer, 2013; Hoermiller *et al*., 2017). Briefly, approximately 100–150 mg of freeze‐dried leaf tissue was suspended in 10 ml heptane‐tetrachlorethylene ρ = 1.34 g cm^−3^ and sonicated for 5 sec with pauses of 15 sec over a time course of 12 min (Branson Sonifier 250, output control 4; Branson, USA). The sonicated suspension was passed through a nylon gauze of 30‐μm pore size and centrifuged afterwards. The pellet was suspended in heptane‐tetrachlorethylene and loaded onto a linear gradient of heptane‐tetrachlorethylene (ρ = 1.34 g cm^−3^) to tetrachlorethylene (ρ = 1.6 g cm^−3^). After ultracentrifugation for 3 h at 100 000 ***g***, the gradient was fractionated into nine fractions that were divided into five subfractions and dried under vacuum. Subfractions were used for enzyme activity measurements, proteomics and metabolomics analysis (one subfraction for each of the assays described in the following paragraphs). The surplus of subfractions was kept as a backup.

### Enzyme activity measurements

Marker enzyme activity analysis for compartments in gradients was performed as described before (Knaupp *et al*., 2011; Nägele and Heyer, 2013). Alkaline pyrophosphatase was used as a plastidial marker, UGPase as a cytosolic marker and acid phosphatase as a marker for the vacuolar compartment.

### Extraction and protein analysis via LC‐MS/MS

Dried pellets from fractionated gradients were solubilized in 8 m urea 50 mm Hepes KOH (pH = 7.8) on ice. Samples were precipitated in acetone with 0.5% β‐mercaptoethanol. Afterwards pellets were washed two times with methanol and acetone, and again solubilized in 8 m urea, 50 mm Hepes KOH (pH = 7.8). Bio‐Rad Bradford assay and BSA as the standard were used to determine protein concentrations. Equal amounts of protein (15 μg) were reduced with dithiothreitol (DTT) at a concentration of 5 mm for 45 min at 37°C, and alkylated at a concentration of 10 mm with iodoacetamide and incubated in the dark for 60 min at 23°C. Alkylation was stopped by increasing the DTT concentration to 10 mm and by incubation in the dark for 15 min. Samples were diluted two‐fold with 20% acetonitrile (ACN) and 100 mm ammonium bicarbonate (AMBIC, Sigma‐Aldrich, St. Louis, MO, USA), proteins were predigested with Lys‐C (1:1000 w:w, Sigma‐Aldrich) at 30°C for 2.5 h in the dark. Samples were diluted two‐fold with 2 m urea and 10% ACN, 25 mm AmBic, 10 mm CaCl_2_ and digested with sequencing grade modified trypsin (Poroszyme, immobilized trypsin; 1:100 v:w) for 12 h. Digested proteins were acidified with formic acid (pH ~3.0), desalted with C18 materials (Bond Elut SPEC, Agilent, Santa Clara, CA, USA) and dried in a vacuum concentrator (ScanVac, LaboGene). Peptides were dissolved in 2% ACN, 0.1% formic acid and the same amount of total protein was loaded and separated on a PepMap RSLC 75‐μm, 50‐cm column (Thermo Fisher Scientific Inc., Waltham, USA). Flow rate was set to 300 nL min^−1^ with 2–40% in 90 min of mobile phase B (mobile phase A: 0.1% formic acid (FA) in water [v/v]; mobile phase B: 0.1% FA in 90% ACN [v/v]). The run ended with 60 min of equilibration. Subsequently, mobile phase B was set from 40 to 90% for 1 min and held stable at 90% for 5 min, followed by continuous decrease to 2%. MS analysis was performed using Orbitrap Elite and Q Exactive instruments (Thermo Fisher Scientific Inc.) in positive mode and a full scan in FT with a resolution of 60,000 in profile mode. Precursor masses ranged between 360–1800 m/z. MS/MS was executed in the linear ion trap with CID fragmentation for the 20 most intense ions, by a minimal signal threshold of 500 counts. Prediction of ion injection time was enabled (5 × 10^2^ ions for up to 10 msec). Dynamic exclusion was enabled with repeat count 1 and a repeat duration of 30 sec. Exclusion list size was set to 500 and exclusion duration to 30 sec. Excluded mass was set to ±10 ppm relative to reference mass, early expiration was enabled with a 1 count and s/n threshold of 2.0.

Peptide identification as well as protein quantification was performed using MaxQuant software (http://www.maxquant.org) and implemented algorithms of version 1.5.5.1 (Cox and Mann, 2008) against TAIR10 (www.arabidopsis.org) protein database (Lamesch *et al*., 2012). Protein analysis for label‐free quantification was carried out with main settings as recommended and a false discovery rate of 0.01. A maximum of two missed cleavages was applied. Maximal five variable modifications per peptide were allowed for N‐terminal acetylation and methionine oxidation carbamidomethylation was set as a fixed modification due to previous methylation. Identification rules for proteins were set by a required minimum of two peptides and two minimum razor + unique peptides. Advanced identification mode was exerted with second peptides search and match between runs, the match window was set to 0.7 min and alignment time window to 20 min. MaxQuant LFQ protein output was normalized to total protein amount per fraction. Whole gradients were additionally normalized to inserted dry weight. Mass spectrometry proteomics data have been deposited in the ProteomeXchange Consortium via the PRIDE (Perez‐Riverol *et al*., 2019) partner repository with the dataset identifier PXD013646.

### Subcellular marker protein data set

Identified proteins were analyzed regarding their subcellular localization with SUBA3 (Tanz *et al*., 2012) and SUBACON (Hooper *et al*., 2014). Proteins were considered as subcellular markers if database entries consisted of confirmed fluorescence protein information solely in one compartment. Additionally, annotator database entries within SUBA3 represented the same unique subcellular localization as fluorescence proteins. Proteins were used as subcellular markers if they were present in the whole dataset, i.e. across all genotypes and conditions. A full list of protein markers is provided in Table S1. Each gradient was analyzed individually for the distribution of subcellular protein markers. Mean relative distributions of gradients and their measured marker enzyme are represented in Figure S1. For each gradient fraction, the mean protein abundance of all selected compartment markers was built and applied to calculate the relative distribution of metabolites.

### Extraction and analysis of primary metabolites by GC‐MS TOF

Primary metabolite amount was quantified with gas chromatography coupled to time‐of‐flight mass spectrometry. Fractionated gradients were extracted as previously described (Weiszmann *et al*., 2018). In brief, samples were extracted twice with methanol:chloroform:water (MCW, 5:2:1 v:v:v), followed by an extraction step with 80% ethanol in which the pellet was heated up to 80°C for 30 min. For phase separation, water was added to the MCW supernatant, and the polar phase was merged with the ethanol extract and dried in a vacuum concentrator (ScanVac, LaboGene). The dried extracts were derivatized applying methoximation (methoxyamine hydrochloride in pyridine) by incubation for 90 min at 30°C. For silylation, *N*‐methyl‐*N*‐(trimethylsilyl)trifluoroacetamide was used and samples were incubated for 30 min at 37°C. Derivatized samples were transferred into glass vials, which were sealed with a crimp cap. GC‐TOF‐MS analysis was performed on an Agilent 6890 gas chromatograph (Agilent Technologies^®^, Santa Clara, USA) coupled to a LECO Pegasus^®^ GCxGC‐TOF mass spectrometer (LECO Corporation, St. Joseph, USA). Compounds were separated on an Agilent column HP5MS (length: 30 m, diameter: 0.25 mm, film: 0.25 μm). Deconvolution of the total ion chromatogram and peak integration was performed using the software LECO Chromatof^®^. Within gradients, relative distribution of metabolites was determined. For absolute quantification, dried nonfractionated plant material was extracted as described above, and calibration curves of six different concentrations within the linear range of detection were used.

### Determination of subcellular metabolite distributions

Subcellular metabolite distribution of each fractionated sample was calculated as described previously (Fürtauer *et al*., 2016). The algorithm applied compared the distribution of marker proteins and metabolites between all subfractions of one sample. To prevent overestimation of technical errors introduced by LC‐MS/MS and GC‐MS quantification of proteins and metabolites, only those subcellular fractions were correlated that differed ≥10% in their normalized abundance of marker proteins and ≥5% in relative metabolite abundance, i.e. peak areas. These protein−metabolite correlations revealed the relative distribution of metabolites across cellular compartments. Comparing subcellular metabolite distributions calculated with and without the 10% and 5% threshold indicated a slight reduction of compartment separation by the filter settings due to the omission of statistically weak protein marker dynamics (Figure S2).

Absolute whole cell metabolite levels were determined from nonfractionated plant material. Absolute subcellular metabolite levels were calculated by multiplication of each relative distribution with each absolute amount. Therefore, multiplication was performed for each relative distribution (three replicates) and each absolute amount (five replicates).

### Statistical analysis and proteome correlation

Statistical analysis was performed using the R software package (The R Project for Statistical Computing; http://www.r-project.org/) (R Core Team, 2017), Microsoft Excel^®^ (www.microsoft.com), and the numerical software environment Matlab^®^ (http://www.mathworks.com).

## Data Statement

All data generated and used in this study are available upon request or as Supporting Information of the article.

## Author Contributions

LF, LK performed experiments, data evaluation, wrote the manuscript. WW supported metabolomics and proteomics analysis. TN and AGH conceived the study, performed the data evaluation and wrote the manuscript.

## Conflict of Interest

The authors declare no conflict of interest.

## Supporting information


**Figure S1**. Reproducibility of NAF gradients among genotypes and time points.

**Figure S2**. Effect of a threshold for marker dynamics on subcellular metabolite distribution.

**Figure S3**. Heterogeneity of LC‐MS/MS determined marker within the representative gradient of Figure 3.

**Figure S4**. Pyruvate and TCA cycle intermediates in all compartments.

**Figure S5**. Subcellular amino acid amount.

**Figure S6**. Venn diagram of high abundant protein quartiles in L*er* (blue) and *gin2‐1* (yellow) after 4 h in the light.

**Figure S7**. Estimated hexose amount in a 4‐compartment model.

**Figure S8**. Plants of L*er* and *gin2‐1* at sampling stage.

**Table S1**. Subcellular protein marker set.

**Table S2**. Pearson correlated subcellular marker set with total proteome.

**Table S3**. Significant metabolic shifts to mitochondria from a three‐compartment model to a four‐compartment model.

**Table S4**. Mean values of primary metabolites in three compartments and *t*‐test results.

**Table S5**. Mean values of primary metabolites in four compartments and *t*‐test results.

## References

[tpj14472-bib-0001] Aguilera‐Alvarado, G.P. and Sánchez‐Nieto, S. (2017) Plant hexokinases are multifaceted proteins. Plant Cell Physiol. 58, 1151–1160. 28449056 10.1093/pcp/pcx062

[tpj14472-bib-0002] Arrivault, S. , Guenther, M. , Florian, A. * **et al.** * (2014) Dissecting the subcellular compartmentation of proteins and metabolites in Arabidopsis leaves using non‐aqueous fractionation. Mol. Cell Proteomics, 13, 2246–2259. 24866124 10.1074/mcp.M114.038190 PMC4159647

[tpj14472-bib-0003] Bermejo, C. , Haerizadeh, F. , Takanaga, H. , Chermak, D. and Frommer, W.B. (2011) Optical sensors for measuring dynamic changes of cytosolic metabolite levels in yeast. Nat. Protoc. 6, 1806. 22036883 10.1038/nprot.2011.391

[tpj14472-bib-0004] Brauner, K. , Stutz, S. , Paul, M. and Heyer, A.G. (2015) Measuring whole plant CO2 exchange with the environment reveals opposing effects of the gin2–1 mutation in shoots and roots of *Arabidopsis thaliana* . Plant Signal. Behav. 10, e973822. 25482780 10.4161/15592324.2014.973822 PMC4623098

[tpj14472-bib-0005] Büssis, D. , Heineke, D. , Sonnewald, U. , Willmitzer, L. , Raschke, K. and Heldt, H.W. (1997) Solute accumulation and decreased photosynthesis in leaves of potato plants expressing yeast‐derived invertase either in the apoplast, vacuole or cytosol. Planta, 202, 126–136. 9177057 10.1007/s004250050111

[tpj14472-bib-0006] Claeyssen, E. and Rivoal, J. (2007) Isozymes of plant hexokinase: occurrence, properties and functions. Phytochemistry, 68, 709–731. 17234224 10.1016/j.phytochem.2006.12.001

[tpj14472-bib-0007] Claros, M.G. and Vincens, P. (1996) Computational method to predict mitochondrially imported proteins and their targeting sequences. Eur. J. Biochem. 241, 779–786. 8944766 10.1111/j.1432-1033.1996.00779.x

[tpj14472-bib-0008] Cox, J. and Mann, M. (2008) MaxQuant enables high peptide identification rates, individualized p.p.b.‐range mass accuracies and proteome‐wide protein quantification. Nat. Biotechnol. 26, 1367. 19029910 10.1038/nbt.1511

[tpj14472-bib-0009] De Col, V. , Fuchs, P. , Nietzel, T. * **et al.** * (2017) ATP sensing in living plant cells reveals tissue gradients and stress dynamics of energy physiology. eLife, 6, e26770. 28716182 10.7554/eLife.26770 PMC5515573

[tpj14472-bib-0010] Depaoli, M.R. , Karsten, F. , Madreiter‐Sokolowski, C.T. * **et al.** * (2018) Real‐time imaging of mitochondrial ATP dynamics reveals the metabolic setting of single cells. Cell Rep. 25, 501–512.e503. 30304688 10.1016/j.celrep.2018.09.027 PMC6456002

[tpj14472-bib-0011] Diekmann, Y. and Pereira‐Leal, J.B. (2013) Evolution of intracellular compartmentalization. Biochem. J. 449, 319–331. 23240612 10.1042/BJ20120957

[tpj14472-bib-0012] Dietz, K.‐J. (2017) Subcellular metabolomics: the choice of method depends on the aim of the study. J. Exp. Bot. 68, 5695–5698. 29155967 10.1093/jxb/erx406 PMC5854114

[tpj14472-bib-0013] Eisenhut, M. , Planchais, S. , Cabassa, C. , Guivarc'h, A. , Justin, A.M. , Taconnat, L. , Renou, J.P. , Linka, M. , Gagneul, D. and Timm, S. (2013) Arabidopsis A BOUT DE SOUFFLE is a putative mitochondrial transporter involved in photorespiratory metabolism and is required for meristem growth at ambient CO 2 levels. Plant J. 73, 836–849. 23181524 10.1111/tpj.12082

[tpj14472-bib-0014] Elbers, R. , Heldt, H.W. , Schmucker, P. , Soboll, S. and Wiese, H. (1974) Measurement of the ATP/ADP ratio in mitochondria and in the extramitochondrial compartment by fractionation of freeze‐stopped liver tissue in non‐aqueous media. Hoppe Seylers Z. Physiol. Chem. 355, 378–394. 4154892 10.1515/bchm2.1974.355.1.378

[tpj14472-bib-0015] Emanuelsson, O. , Nielsen, H. and Heijne, G.V. (1999) ChloroP, a neural network‐based method for predicting chloroplast transit peptides and their cleavage sites. Protein Sci. 8, 978–984. 10338008 10.1110/ps.8.5.978 PMC2144330

[tpj14472-bib-0016] Emanuelsson, O. , Brunak, S. , von Heijne, G. and Nielsen, H. (2007) Locating proteins in the cell using TargetP, SignalP and related tools. Nat. Protoc. 2, 953. 17446895 10.1038/nprot.2007.131

[tpj14472-bib-0017] Farré, E.M. , Tiessen, A. , Roessner, U. , Geigenberger, P. , Trethewey, R.N. and Willmitzer, L. (2001) Analysis of the compartmentation of glycolytic intermediates, nucleotides, sugars, organic acids, amino acids, and sugar alcohols in potato tubers using a nonaqueous fractionation method. Plant Physiol. 127, 685–700. 11598242 10.1104/pp.010280 PMC125103

[tpj14472-bib-0018] Field, M.C. and Dacks, J.B. (2009) First and last ancestors: reconstructing evolution of the endomembrane system with ESCRTs, vesicle coat proteins, and nuclear pore complexes. Curr. Opin. Cell Biol. 21, 4–13. 19201590 10.1016/j.ceb.2008.12.004

[tpj14472-bib-0019] Figueroa, C.M. , Feil, R. , Ishihara, H. * **et al.** * (2016) Trehalose 6‐phosphate coordinates organic and amino acid metabolism with carbon availability. Plant J. 85, 410–423. 26714615 10.1111/tpj.13114

[tpj14472-bib-0020] Fürtauer, L. , Weckwerth, W. and Nägele, T. (2016) A benchtop fractionation procedure for subcellular analysis of the plant metabolome. Front. Plant Sci. 7, 1912. 28066469 10.3389/fpls.2016.01912 PMC5177628

[tpj14472-bib-0021] Gauthier, P.P. , Bligny, R. , Gout, E. , Mahé, A. , Nogués, S. , Hodges, M. and Tcherkez, G.G. (2010) In folio isotopic tracing demonstrates that nitrogen assimilation into glutamate is mostly independent from current CO_2_ assimilation in illuminated leaves of *Brassica napus* . New Phytol. 185, 988–999. 20070539 10.1111/j.1469-8137.2009.03130.x

[tpj14472-bib-0022] Gerhardt, R. and Heldt, H.W. (1984) Measurement of subcellular metabolite levels in leaves by fractionation of freeze‐stopped material in nonaqueous media. Plant Physiol. 75, 542–547. 16663663 10.1104/pp.75.3.542 PMC1066952

[tpj14472-bib-0023] Granot, D. , Kelly, G. , Stein, O. and David‐Schwartz, R. (2014) Substantial roles of hexokinase and fructokinase in the effects of sugars on plant physiology and development. J. Exp. Bot. 65, 809–819. 24293612 10.1093/jxb/ert400

[tpj14472-bib-0024] Hanning, I. and Heldt, H.W. (1993) On the function of mitochondrial metabolism during photosynthesis in spinach (Spinacia oleracea L.) leaves (partitioning between respiration and export of redox equivalents and precursors for nitrate assimilation products). Plant Physiol. 103, 1147–1154. 12232008 10.1104/pp.103.4.1147 PMC159100

[tpj14472-bib-0025] Harrington, H.A. , Feliu, E. , Wiuf, C. and Stumpf, M.P. (2013) Cellular compartments cause multistability and allow cells to process more information. Biophys. J. 104, 1824–1831. 23601329 10.1016/j.bpj.2013.02.028 PMC3628565

[tpj14472-bib-0026] Heber, U. (1957) Zur Frage der Lokalisation von löslichen Zuckern in der Pflanzenzelle. Ber. Dtsch. Bot. Ges. 70, 371–382.

[tpj14472-bib-0027] Heber, U. (2002) Irrungen, Wirrungen? The Mehler reaction in relation to cyclic electron transport in C3 plants. Photosynth. Res. 73, 223–231. 16245125 10.1023/A:1020459416987

[tpj14472-bib-0028] Hoermiller, I.I. , Naegele, T. , Augustin, H. , Stutz, S. , Weckwerth, W. and Heyer, A.G. (2017) Subcellular reprogramming of metabolism during cold acclimation in *Arabidopsis thaliana* . Plant, Cell Environ. 40, 602–610. 27642699 10.1111/pce.12836

[tpj14472-bib-0029] Hooper, C.M. , Tanz, S.K. , Castleden, I.R. , Vacher, M.A. , Small, I.D. and Millar, A.H. (2014) SUBAcon: a consensus algorithm for unifying the subcellular localization data of the Arabidopsis proteome. Bioinformatics, 30, 3356–3364. 25150248 10.1093/bioinformatics/btu550

[tpj14472-bib-0030] Huber, S.C. (1989) Biochemical mechanism for regulation of sucrose accumulation in leaves during photosynthesis. Plant Physiol. 91, 656–662. 16667083 10.1104/pp.91.2.656 PMC1062051

[tpj14472-bib-0031] Jaipargas, E.‐A. , Barton, K.A. , Mathur, N. and Mathur, J. (2015) Mitochondrial pleomorphy in plant cells is driven by contiguous ER dynamics. Front. Plant Sci. 6, 783–783. 26442089 10.3389/fpls.2015.00783 PMC4585081

[tpj14472-bib-0032] Jardim‐Messeder, D. , Caverzan, A. , Rauber, R. , Souza Ferreira, E. , Margis‐Pinheiro, M. and Galina, A. (2015) Succinate dehydrogenase (mitochondrial complex II) is a source of reactive oxygen species in plants and regulates development and stress responses. New Phytol. 208, 776–789. 26082998 10.1111/nph.13515

[tpj14472-bib-0033] Knaupp, M. , Mishra, K.B. , Nedbal, L. and Heyer, A.G. (2011) Evidence for a role of raffinose in stabilizing photosystem II during freeze‐thaw cycles. Planta, 234, 477–486. 21533754 10.1007/s00425-011-1413-0

[tpj14472-bib-0034] Küstner, L. , Nägele, T. and Heyer, A.G. (2019) Mathematical modeling of diurnal patterns of carbon allocation to shoot and root in *Arabidopsis thaliana* . NPJ Syst. Biol. Appl. 5, 4. 30701083 10.1038/s41540-018-0080-1 PMC6346032

[tpj14472-bib-0035] Lamesch, P. , Berardini, T.Z. , Li, D. * **et al.** * (2012) The Arabidopsis Information Resource (TAIR): improved gene annotation and new tools. Nucleic Acids Res. 40, D1202–D1210. 22140109 10.1093/nar/gkr1090 PMC3245047

[tpj14472-bib-0036] Lee, C.P. , Eubel, H. and Millar, A.H. (2010) Diurnal changes in mitochondrial function reveal daily optimisation of light and dark respiratory metabolism in Arabidopsis. Mol. Cell Proteomics, 9, 2125–2139. 20601493 10.1074/mcp.M110.001214 PMC2953910

[tpj14472-bib-0037] Li, L. and Sheen, J. (2016) Dynamic and diverse sugar signaling. Curr. Opin. Plant Biol. 33, 116–125. 27423125 10.1016/j.pbi.2016.06.018 PMC5050104

[tpj14472-bib-0038] Li, L. , Shi, L. , Wu, Y. , Sheen, J. , Fu, L. , Xiong, Y. and Liu, Y. (2019) Integration of nutrient, energy, light, and hormone signalling via TOR in plants. J. Exp. Bot. 70, 2227–2238. 30715492 10.1093/jxb/erz028 PMC6463029

[tpj14472-bib-0039] Moore, B. , Zhou, L. , Rolland, F. , Hall, Q. , Cheng, W.‐H. , Hwang, I. , Jones, T. and Sheen, J. (2003) Role of the Arabidopsis glucose sensor HXK1 in nutrient, light, and hormonal signaling. Science, 300, 332–336. 12690200 10.1126/science.1080585

[tpj14472-bib-0040] Nägele, T. and Heyer, A.G. (2013) Approximating subcellular organisation of carbohydrate metabolism during cold acclimation in different natural accessions of *Arabidopsis thaliana* . New Phytol. 198, 777–787. 23488986 10.1111/nph.12201

[tpj14472-bib-0041] Nägele, T. , Henkel, S. , Hörmiller, I. , Sauter, T. , Sawodny, O. , Ederer, M. and Heyer, A.G. (2010) Mathematical modeling of the central carbohydrate metabolism in Arabidopsis reveals a substantial regulatory influence of vacuolar invertase on whole plant carbon metabolism. Plant Physiol. 153, 260–272. 20207708 10.1104/pp.110.154443 PMC2862412

[tpj14472-bib-0042] Noctor, G. , Arisi, A.‐C.M. , Jouanin, L. and Foyer, C.H. (1999) Photorespiratory glycine enhances glutathione accumulation in both the chloroplastic and cytosolic compartments. J. Exp. Bot. 50, 1157–1167.

[tpj14472-bib-0043] Nukarinen, E. , Nagele, T. , Pedrotti, L. * **et al.** * (2016) Quantitative phosphoproteomics reveals the role of the AMPK plant ortholog SnRK1 as a metabolic master regulator under energy deprivation. Sci. Rep. 6, 31697. 27545962 10.1038/srep31697 PMC4992866

[tpj14472-bib-0044] Obata, T. , Florian, A. , Timm, S. , Bauwe, H. and Fernie, A.R. (2016) On the metabolic interactions of (photo)respiration. J. Exp. Bot. 67, 3003–3014. 27029352 10.1093/jxb/erw128

[tpj14472-bib-0045] Perez‐Riverol, Y. , Csordas, A. , Bai, J. * **et al.** * (2019) The PRIDE database and related tools and resources in 2019: improving support for quantification data. Nucleic Acids Res. 47, D442–D450. 30395289 10.1093/nar/gky1106 PMC6323896

[tpj14472-bib-0046] Popov, V.N. , Eprintsev, A.T. , Fedorin, D.N. and Igamberdiev, A.U. (2010) Succinate dehydrogenase in *Arabidopsis thaliana* is regulated by light via phytochrome A. FEBS Lett. 584, 199–202. 19932106 10.1016/j.febslet.2009.11.057

[tpj14472-bib-0047] R Core Team (2017) R: A language and environment for statistical computing. Vienna, Austria. https://www.R-project.org/

[tpj14472-bib-0048] Rolland, F. , Baena‐Gonzalez, E. and Sheen, J. (2006) Sugar sensing and signaling in plants: conserved and novel mechanisms. Annu. Rev. Plant Biol. 57, 675–709. 16669778 10.1146/annurev.arplant.57.032905.105441

[tpj14472-bib-0049] Scheibe, R. (2004) Malate valves to balance cellular energy supply. Physiol. Plant. 120, 21–26. 15032873 10.1111/j.0031-9317.2004.0222.x

[tpj14472-bib-0050] Sheen, J. (1994) Feedback control of gene expression. Photosynth. Res. 39, 427–438. 24311134 10.1007/BF00014596

[tpj14472-bib-0051] Sheen, J. (2014) Master regulators in plant glucose signaling networks. J. Plant Biol. 57, 67–79. 25530701 10.1007/s12374-014-0902-7 PMC4270195

[tpj14472-bib-0052] Stitt, M. and Hurry, V. (2002) A plant for all seasons: alterations in photosynthetic carbon metabolism during cold acclimation in Arabidopsis. Curr. Opin. Plant Biol. 5, 199–206. 11960736 10.1016/s1369-5266(02)00258-3

[tpj14472-bib-0053] Sweetlove, L.J. , Beard, K.F.M. , Nunes‐Nesi, A. , Fernie, A.R. and Ratcliffe, R.G. (2010) Not just a circle: flux modes in the plant TCA cycle. Trends Plant Sci. 15, 462–470. 20554469 10.1016/j.tplants.2010.05.006

[tpj14472-bib-0054] Tanz, S.K. , Castleden, I. , Hooper, C.M. , Vacher, M. , Small, I. and Millar, H.A. (2012) SUBA3: a database for integrating experimentation and prediction to define the SUBcellular location of proteins in Arabidopsis. Nucleic Acids Res. 41, D1185–D1191. 23180787 10.1093/nar/gks1151 PMC3531127

[tpj14472-bib-0055] Tcherkez, G. , Mahé, A. , Gauthier, P. , Mauve, C. , Gout, E. , Bligny, R. , Cornic, G. and Hodges, M. (2009) In folio respiratory fluxomics revealed by 13C isotopic labeling and H/D isotope effects highlight the noncyclic nature of the tricarboxylic acid “cycle” in illuminated leaves. Plant Physiol. 151, 620–630. 19675152 10.1104/pp.109.142976 PMC2754646

[tpj14472-bib-0056] Tiessen, A. , Hendriks, J.H.M. , Stitt, M. , Branscheid, A. , Gibon, Y. , Farré, E.M. and Geigenberger, P. (2002) Starch synthesis in potato tubers is regulated by post‐translational redox modification of ADP‐glucose pyrophosphorylase: a novel regulatory mechanism linking starch synthesis to the sucrose supply. Plant Cell, 14, 2191–2213. 12215515 10.1105/tpc.003640 PMC150765

[tpj14472-bib-0057] Timm, S. , Florian, A. , Wittmiß, M. , Jahnke, K. , Hagemann, M. , Fernie, A. and Bauwe, H. (2013) Serine acts as metabolic signal for the transcriptional control of photorespiration‐related genes in *Arabidopsis thaliana* . Plant Physiol. 162, 379–389. 23471132 10.1104/pp.113.215970 PMC3641216

[tpj14472-bib-0058] Weiszmann, J. , Fürtauer, L. , Weckwerth, W. and Nägele, T. (2018) Vacuolar sucrose cleavage prevents limitation of cytosolic carbohydrate metabolism and stabilizes photosynthesis under abiotic stress. FEBS J. 285, 4082–4098. 30216682 10.1111/febs.14656

[tpj14472-bib-0059] Wingler, A. , Lea, P.J. , Quick, W.P. and Leegood, R.C. (2000) Photorespiration: metabolic pathways and their role in stress protection. Philos. Trans. R. Soc. Lond. B Biol. Sci. 355, 1517–1529. 11128005 10.1098/rstb.2000.0712 PMC1692872

[tpj14472-bib-0060] Xiang, L. , Li, Y. , Rolland, F. and Van den Ende, W. (2011) Neutral invertase, hexokinase and mitochondrial ROS homeostasis: emerging links between sugar metabolism, sugar signaling and ascorbate synthesis. Plant Signal. Behav. 6, 1567–1573. 21918379 10.4161/psb.6.10.17036 PMC3256386

[tpj14472-bib-0061] Xiao, W. , Sheen, J. and Jang, J.‐C. (2000) The role of hexokinase in plant sugar signal transduction and growth and development. Plant Mol. Biol. 44, 451–461. 11197321 10.1023/a:1026501430422

[tpj14472-bib-0062] Zhang, Z. , Chen, W. , Zhao, Y. and Yang, Y. (2018) Spatiotemporal imaging of cellular energy metabolism with genetically‐encoded fluorescent sensors in Brain. Neurosci. Bull. 34, 875–886. 29679217 10.1007/s12264-018-0229-3 PMC6129245

