# Supplementary figures and images for: Resolving subcellular plant metabolism
Source: Plant J. 2019 Sep 25;100(3):438–55. doi: 10.1111/tpj.14472 (PMC8653894; doi:10.1111/tpj.14472)

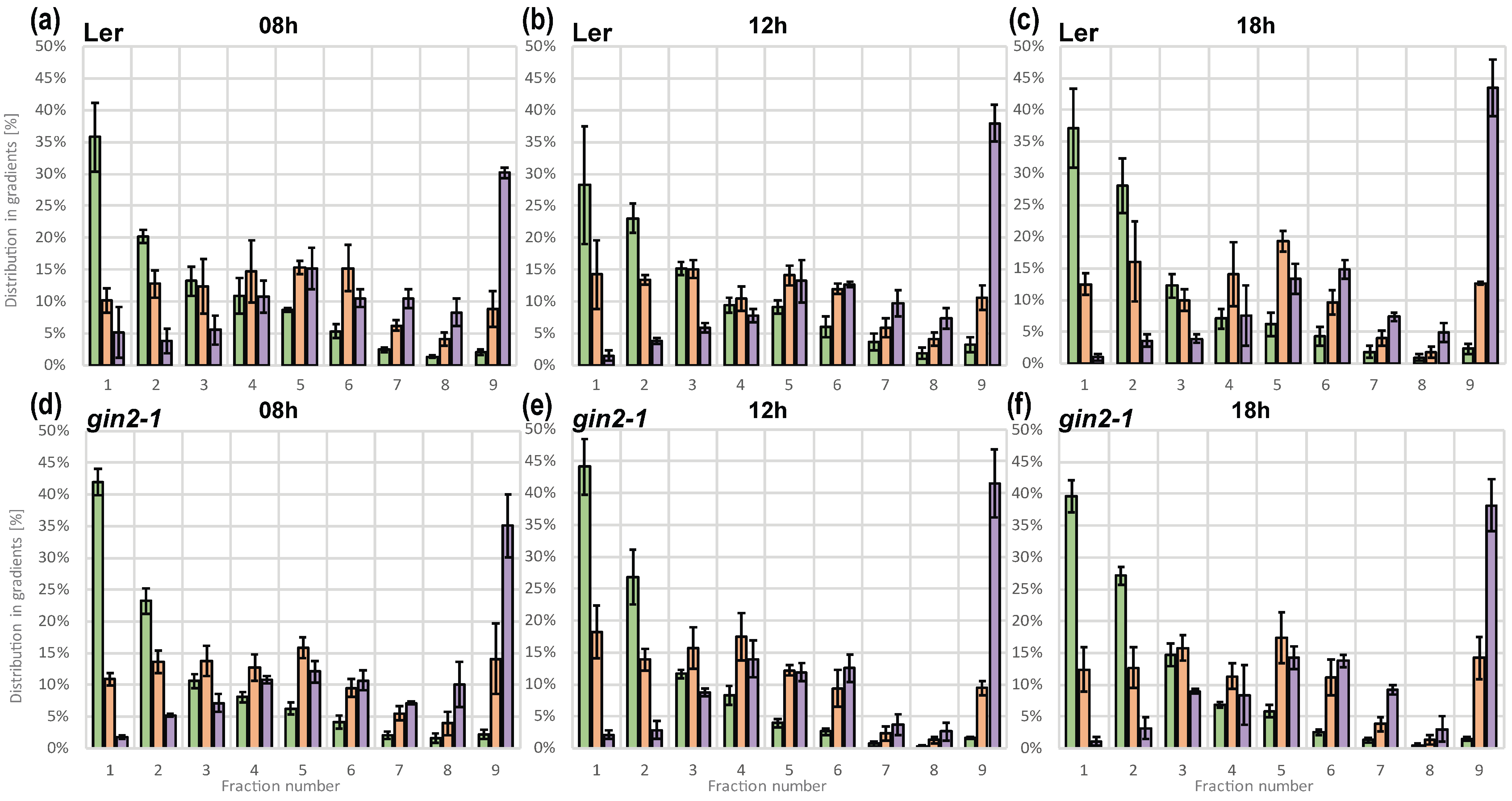

Supplement: Supplementary file 1 — Figure S1. Reproducibility of NAF gradients among genotypes and time points. [file TPJ-100-438-s010.tif]

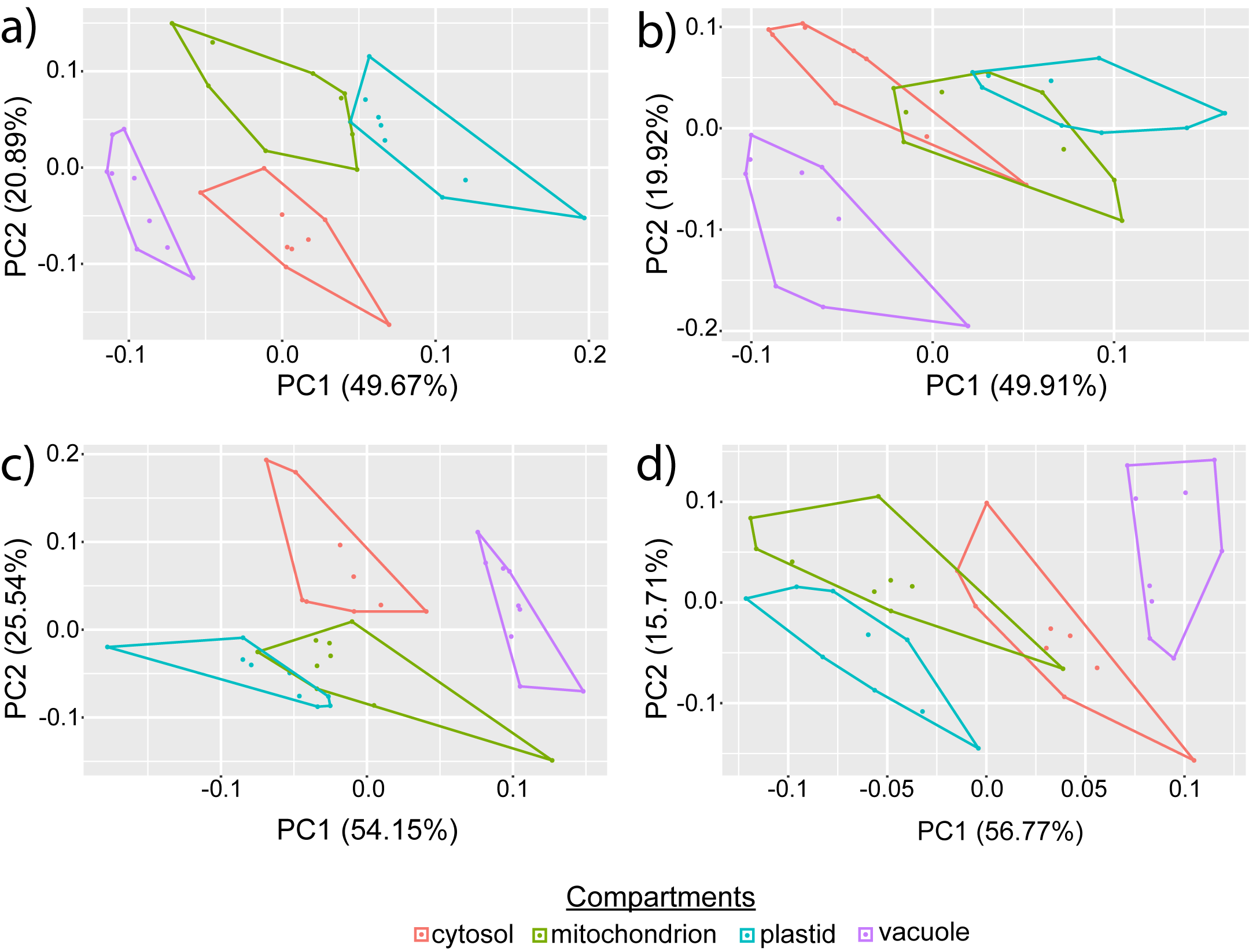

Supplement: Supplementary file 2 — Figure S2. Effect of a threshold for marker dynamics on subcellular metabolite distribution. [file TPJ-100-438-s012.tif]

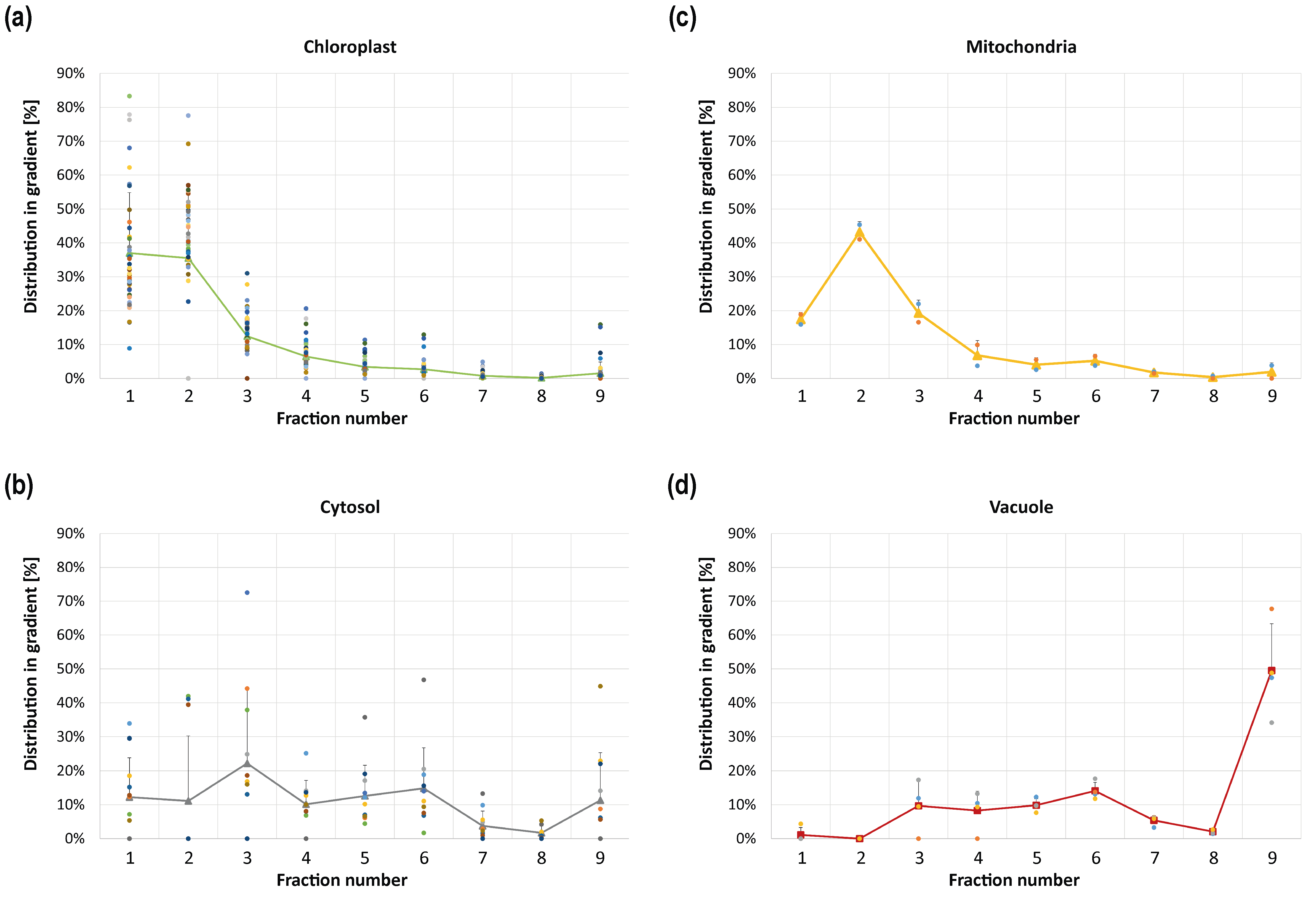

Supplement: Supplementary file 3 — Figure S3. Heterogeneity of LC‐MS/MS determined marker within the representative gradient of Figure 3. [file TPJ-100-438-s001.tif]

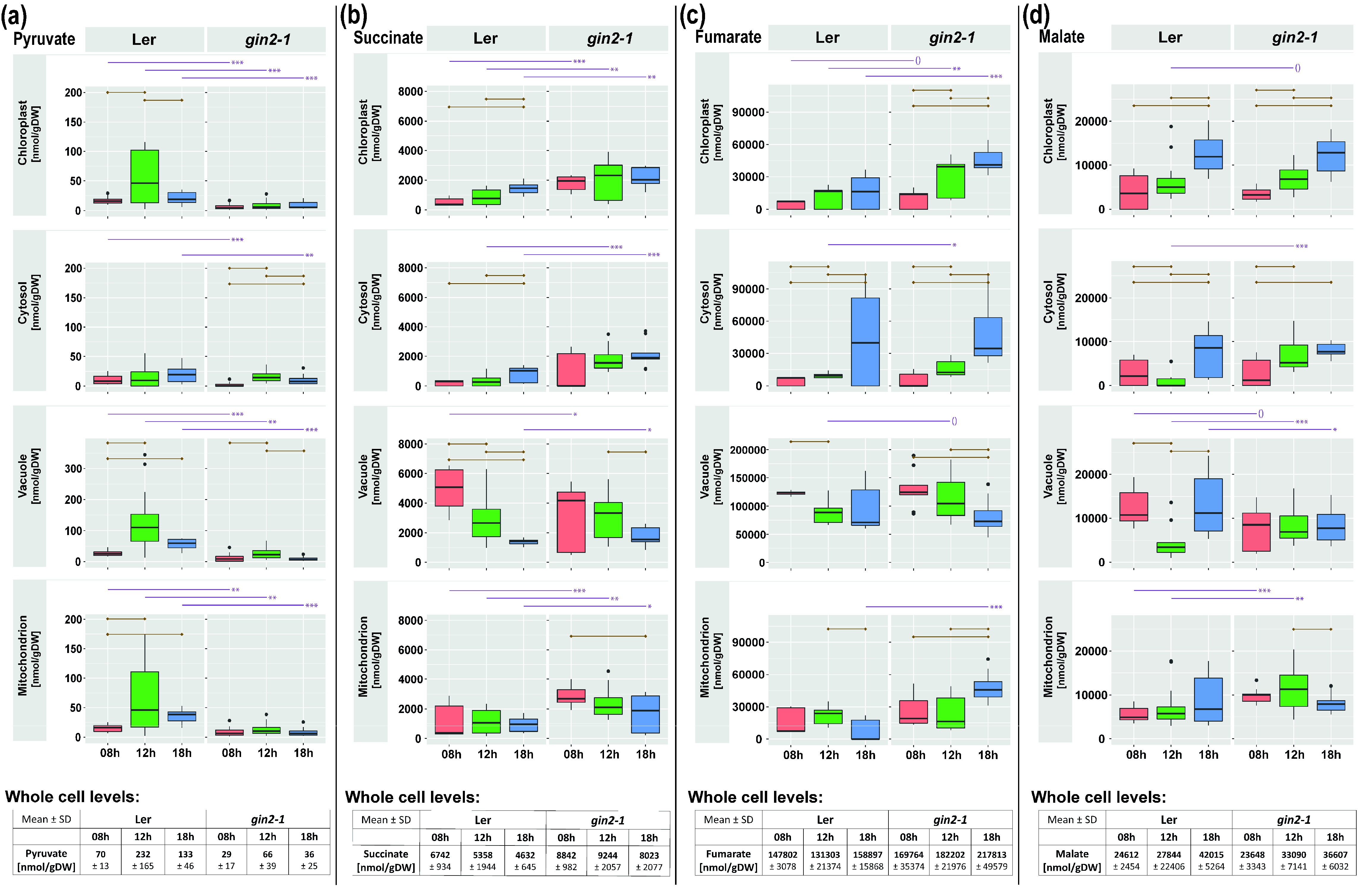

Supplement: Supplementary file 4 — Figure S4. Pyruvate and TCA cycle intermediates in all compartments. [file TPJ-100-438-s002.tif]

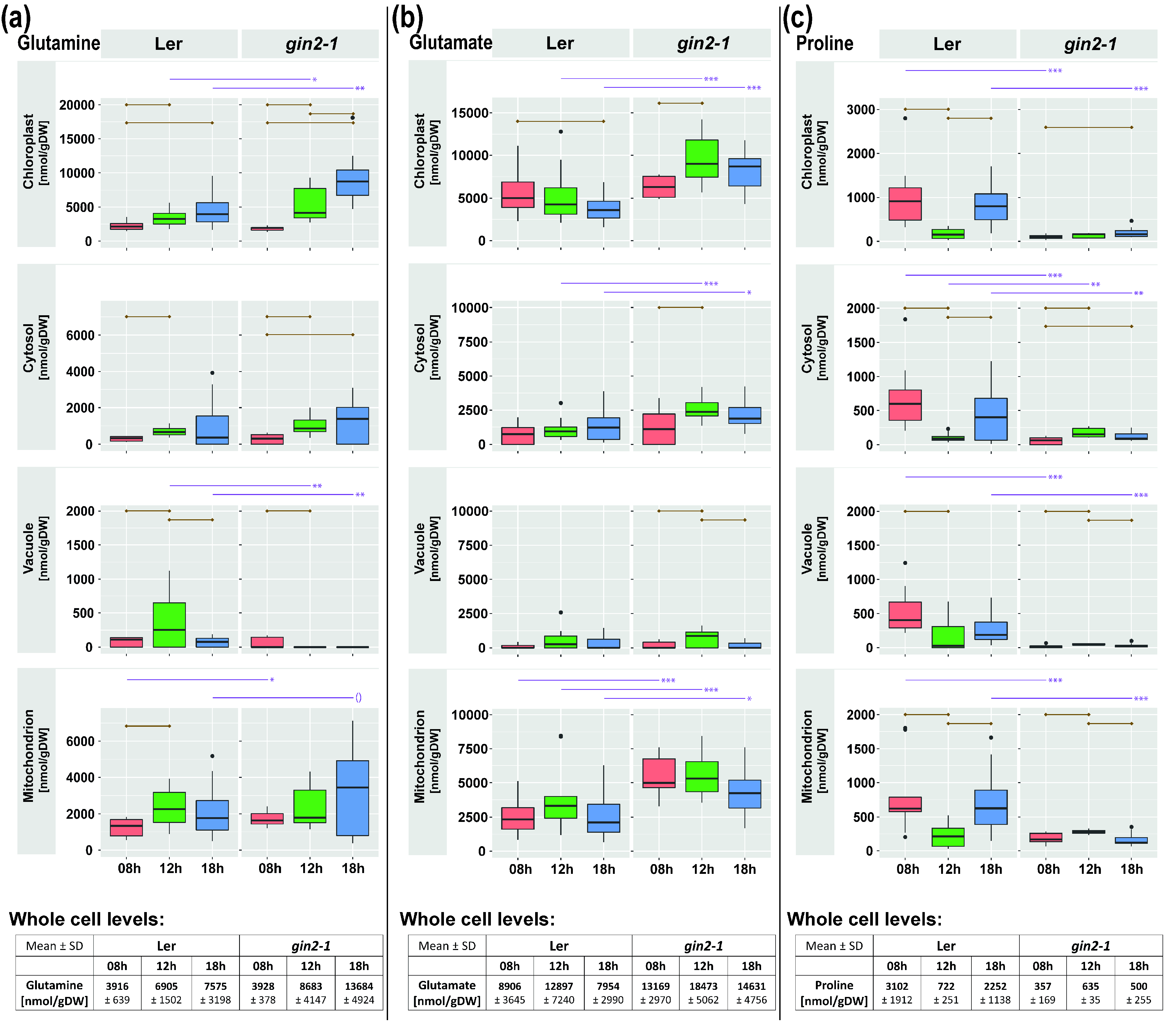

Supplement: Supplementary file 5 — Figure S5. Subcellular amino acid amount. [file TPJ-100-438-s003.tif]

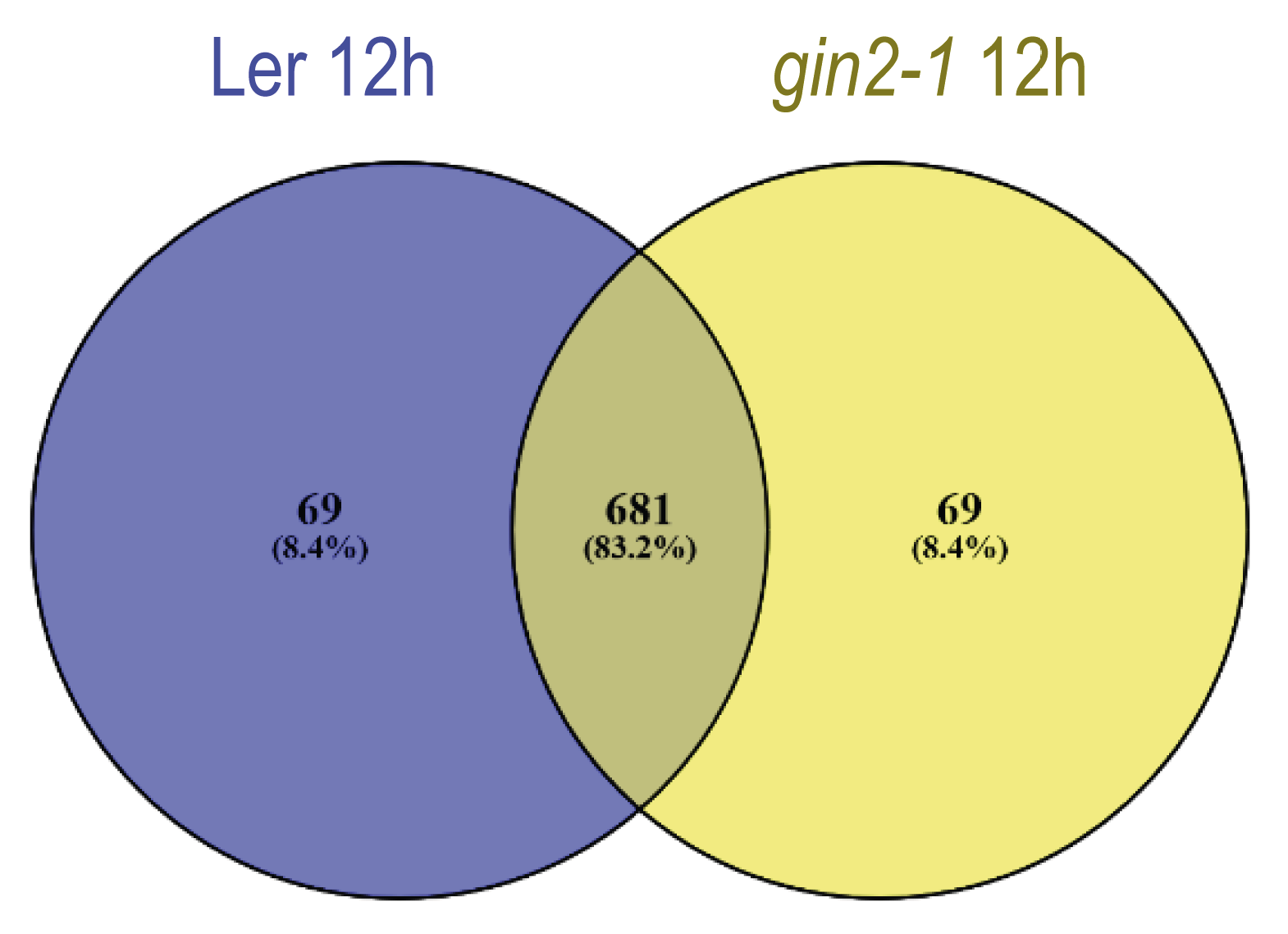

Supplement: Supplementary file 6 — Figure S6. Venn diagram of high abundant protein quartiles in Ler (blue) and gin2‐1 (yellow) after 4 h in the light. [file TPJ-100-438-s004.tif]

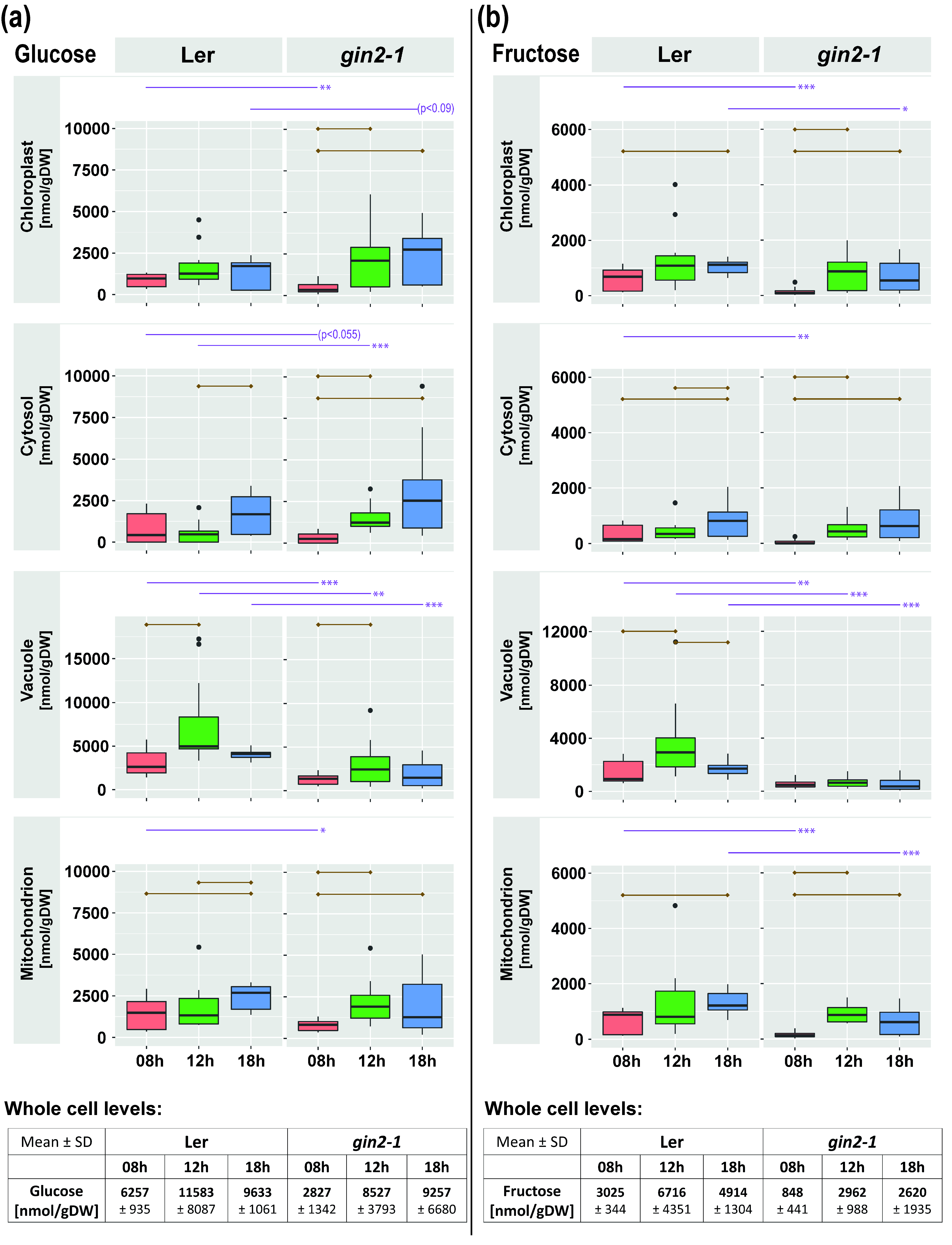

Supplement: Supplementary file 7 — Figure S7. Estimated hexose amount in a 4‐compartment model. [file TPJ-100-438-s005.tif]

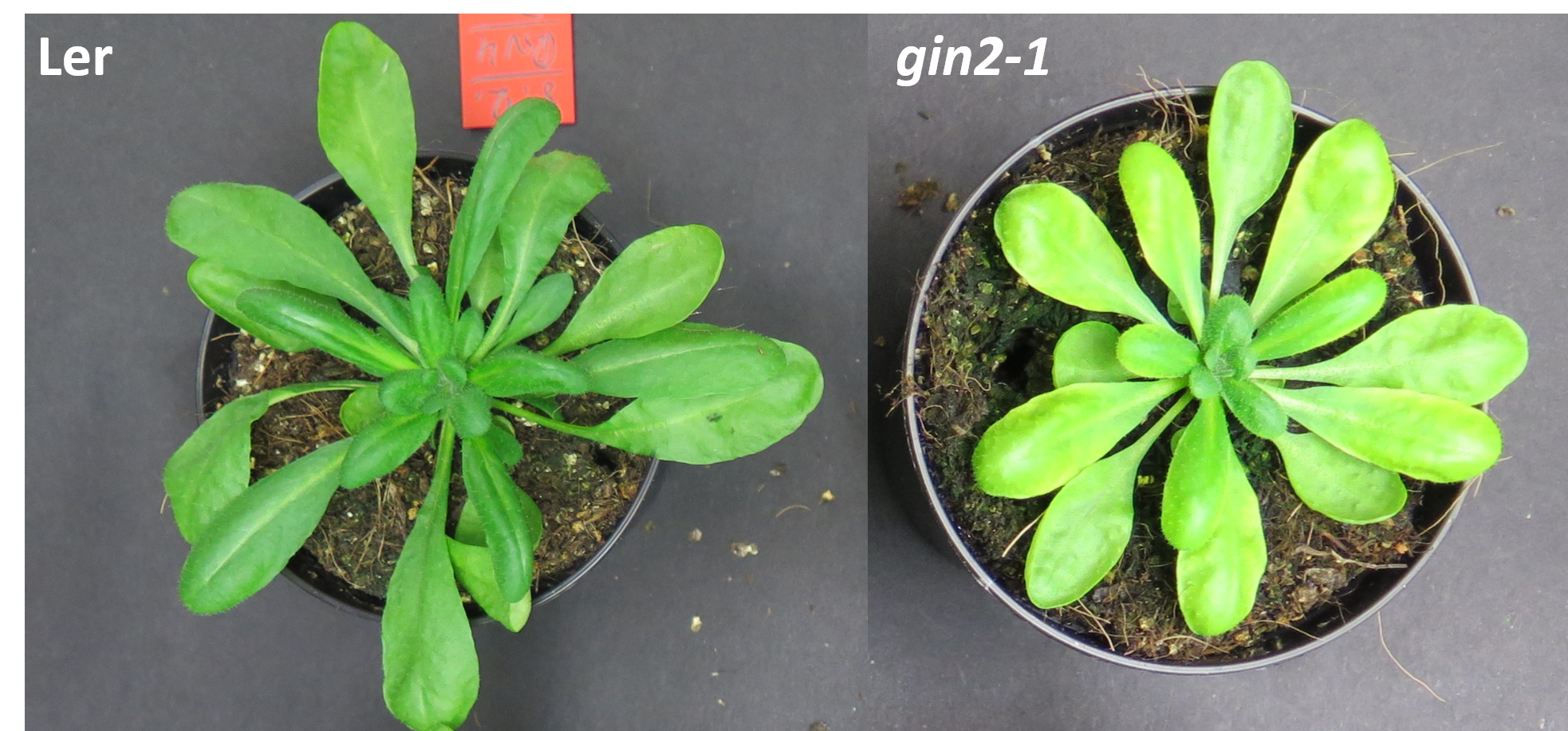

Supplement: Supplementary file 8 — Figure S8. Plants of Ler and gin2‐1 at sampling stage. [file TPJ-100-438-s006.png]
