# Supplementary material for: Resolving subcellular plant metabolism
Source: Plant J. 2019 Sep 25;100(3):438–55. doi: 10.1111/tpj.14472 (PMC8653894; doi:10.1111/tpj.14472)
Supplement: Supplementary file 9 — Table S1. Subcellular protein marker set. [file TPJ-100-438-s007.docx]

| Marker localization | ID |  | Marker localization | ID |
| --- | --- | --- | --- | --- |
| cytosol | AT1G52340.1 |  | **plastid** | AT4G09020.1 |
| cytosol | AT1G74090.1 |  | **plastid** | AT4G11175.1 |
| cytosol | AT1G74100.1 |  | **plastid** | AT4G12060.1 |
| cytosol | AT2G40840.1 |  | **plastid** | AT4G16155.1 |
| cytosol | AT2G45240.1 |  | **plastid** | AT4G17040.1 |
| cytosol | AT3G19710.1 |  | **plastid** | AT4G17090.1 |
| cytosol | AT3G25530.1 |  | **plastid** | AT4G25370.1 |
| cytosol | AT4G39800.1 |  | **plastid** | AT4G26555.1 |
| cytosol | AT5G14800.1 |  | **plastid** | AT4G34120.1 |
| cytosol | AT5G44070.1 |  | **plastid** | AT4G34620.1 |
| cytosol | AT5G49650.1 |  | **plastid** | AT4G35630.1 |
| mitochondrion | AT3G07770.1 |  | **plastid** | AT4G39960.1 |
| mitochondrion | AT4G26780.1 |  | **plastid** | AT5G13410.1 |
| nucleus | AT1G07370.1 |  | **plastid** | AT5G16710.1 |
| nucleus | AT1G48920.1 |  | **plastid** | AT5G45390.1 |
| nucleus | AT2G30620.1 |  | **plastid** | AT5G48300.1 |
| nucleus | AT5G04280.1 |  | **plastid** | AT5G55280.1 |
| plastid | AT1G03475.1 |  | **plastid** | AT5G62790.1 |
| plastid | AT1G12800.1 |  | **vacuole** | AT1G12240.1 |
| plastid | AT1G17650.1 |  | **vacuole** | AT1G62660.1 |
| plastid | AT1G23740.1 |  | **vacuole** | AT5G23820.1 |
| plastid | AT1G29700.1 |  | **vacuole** | AT5G60360.1 |
| plastid | AT1G49970.1 |  | **peroxisome** | AT1G60550.1 |
| plastid | AT1G50900.1 |  | **peroxisome** | AT3G02360.2 |
| plastid | AT1G63610.1 |  |  |  |
| plastid | AT1G65260.1 |  |  |  |
| plastid | AT1G69830.1 |  |  |  |
| plastid | AT1G71500.1 |  |  |  |
| plastid | AT1G74920.1 |  |  |  |
| plastid | AT1G80600.1 |  |  |  |
| plastid | AT2G02500.1 |  |  |  |
| plastid | AT2G20890.1 |  |  |  |
| plastid | AT2G43100.1 |  |  |  |
| plastid | AT2G43560.1 |  |  |  |
| plastid | AT2G45300.1 |  |  |  |
| plastid | AT3G01500.1 |  |  |  |
| plastid | AT3G02730.1 |  |  |  |
| plastid | AT3G08740.1 |  |  |  |
| plastid | AT3G10060.1 |  |  |  |
| plastid | AT3G10620.1 |  |  |  |
| plastid | AT3G22960.1 |  |  |  |
| plastid | AT3G47070.1 |  |  |  |
| plastid | AT3G60370.1 |  |  |  |
| plastid | AT4G01940.1 |  |  |  |
| plastid | AT4G03520.1 |  |  |  |
| plastid | AT4G04770.1 |  |  |  |
