# Supplementary material for: Resolving subcellular plant metabolism
Source: Plant J. 2019 Sep 25;100(3):438–55. doi: 10.1111/tpj.14472 (PMC8653894; doi:10.1111/tpj.14472)
Supplement: Supplementary file 11 — Table S3. Significant metabolic shifts to mitochondria from a three‐compartment model to a four‐compartment model. [file TPJ-100-438-s009.docx]

**Supporting Table S3:** Significant metabolic shifts to mitochondria from a 3-compartment model to a 4-compartment model. Primary metabolites were shifted significantly from indicated compartments to mitochondria. First column: changes in significances between genotypes at the same time point. Second and third column: Significant changes within genotypes and time points (Bonferroni corrected; p<0.05). ‘+’ indicates a simultaneous shift, ‘/’ indicates a shift at different time points.

| Metabolite | Between genotypes | Ler | *gin2-1* |
| --- | --- | --- | --- |
| Alanine |  |  | Vacuole |
| Asparagine |  | Chloroplast/ Vacuole |  |
| Aspartate |  |  |  |
| Citrate | Chloroplast |  | Chloroplast |
| Fructose |  |  |  |
| Fumarate |  |  |  |
| Galactinol |  |  |  |
| Gluconate |  |  |  |
| Glucose | Cytosol | Chloroplast |  |
| Glutamate | Chloroplast/Vacuole |  |  |
| Glutamine |  |  |  |
| Glycine |  |  |  |
| Isoleucine |  | Vacuole |  |
| Leucine |  |  | Vacuole |
| Lysine |  |  |  |
| Malate | Chloroplast+Vacuole |  |  |
| Maltose | Chloroplast |  | Vacuole |
| Melibiose |  | Cytosol | Vacuole |
| Methionine |  |  |  |
| Ornithine | Vacuole |  |  |
| Phenylalanine | Chloroplast |  | Chloroplast/ Chloroplast |
| Proline |  |  | Cytosol |
| Putrescine |  |  | Chloroplast |
| Pyruvate |  |  |  |
| Raffinose |  | Chloroplast |  |
| Serine |  | Chloroplast |  |
| Spermidine | Chloroplast |  |  |
| Succinate |  |  |  |
| Sucrose |  | Vacuole | Vacuole |
| Threitol | Cytosol |  | Cytosol |
| Threonate | Chloroplast/Vacuole |  |  |
| Threonine |  |  | Chloroplast+Vacuole/ Vacuole |
| Tryptophan | Chloroplast |  |  |
| Tyrosine |  |  |  |
| Valine |  |  |  |
| myo-Inositol |  |  | Chloroplast |
