# Supplementary material for: Resolving subcellular plant metabolism
Source: Plant J. 2019 Sep 25;100(3):438–55. doi: 10.1111/tpj.14472 (PMC8653894; doi:10.1111/tpj.14472)
Supplement: Supplementary file 14 [file TPJ-100-438-s011.docx]

Supporting Information

**Fig. S1**: **Reproducibility of NAF gradients among genotypes and time points.** **(a-c)** Ler and **(d-f)** *gin2-1* with defined protein marker set (green: chloroplast, orange: cytosol, purple: vacuole) for time points 8h **(a,d)**, 12h **(b,e)**, 18h **(c,f)** (Mean ± SEM, n=3). Marker protein lists are provided in Supplemental Table S1.

**Fig. S2: Effect of a threshold for marker dynamics on subcellular metabolite distribution. a)** Ler without threshold, **b)** Ler with threshold, **c)** *gin2-1* without threshold, **d)** *gin2-1* with threshold of 10% for proteins and 5% for metabolites. To prevent overestimation of technical errors introduced by LC-MS/MS and GC-MS quantification of proteins and metabolites, only those subcellular fractions were correlated which differed ≥10 % in their normalized abundance of marker proteins and ≥5 % in relative metabolite abundance, i.e. peak areas.

**Fig. S3: Heterogeneity of LC-MS/MS determined marker within the representative gradient of Fig 3.** Every dot represents a marker and its relative distribution within a gradient. Continuous lines connect mean values of relative marker intensities between fractions. Mean values of relative marker intensities were used for correlations with metabolites. **(a)** chloroplast (total number of markers: 47), **(b)** cytosol (total number of markers: 11), **(c)** mitochondria (total number of markers: 2) and **(d)** vacuole (total number of markers: 4).

**Fig. S4: Pyruvate and TCA cycle intermediates in all compartments. (a)** Pyruvate, **(b)** Succinate, **(c)** Fumarate and **(d)** Malate in [nmol/gDW] for both genotypes. Purple lines above graphs indicate significant differences between genotypes at the same time point (n=3 x 5 = 15, t-Test, () p<0.1, * p<0.05, ** p<0.01, *** p<0.001). Brown lines inside of graphs indicate significant differences within genotypes (t-Test, p<0.05, Bonferroni correction of multiple comparisons). Tables below graphs contain absolute metabolite amount at a whole cell level (mean ± SD, n=5).

**Fig. S5: Subcellular amino acid amount. (a)** Glutamine **(b)** Glutamate **(c)** Proline in [nmol/gDW] for both genotypes. Purple lines above graphs indicate significant differences between genotypes at the same time point (n=3 x 5 = 15, t-Test, () p<0.1, * p<0.05, ** p<0.01, *** p<0.001). Brown lines inside of graphs indicate significant differences within genotypes (t-Test, p<0.05, Bonferroni correction of multiple comparisons). Tables below graphs contain absolute metabolite amount at a whole cell level (mean ± SD, n=5).

**Fig. S6:** **Venn-diagram of high abundant protein quartiles in Ler (blue) and *gin2-1* (yellow) after 4h in the light.** All proteins were listed in descending order of their abundance in Ler and *gin2-1*. The 25% with highest proteins abundance were subjected to Venn analysis. 54% of the 69 proteins which were contained in the quartile of Ler but not in *gin2-1* had significantly higher protein levels. *Vice versa*, 44% of the 69 proteins which were contained in the quartile of *gin2-1* but not of Ler had significantly higher protein levels (t-test, p<0.05).

**Fig. S7: Estimated hexose amount in a 4-compartment model.** **(a)** Glucose and **(b)** Fructose in [nmol/gDW] for chloroplast, cytosol, vacuole and mitochondria. Purple lines above graphs indicate a significant difference between genotypes at the same time point (n=3 x 5 = 15, t-Test, * p<0.05, ** p<0.01, *** p<0.001). Brown lines inside of graphs indicate a significant difference between time points (t-Test, p<0.05, Bonferroni correction of multiple comparisons). Tables below graphs contain absolute amount of metabolites at a whole cell level (mean ± SD, n=5).

**Fig. S8: Plants of Ler and *gin2-1* at sampling stage.** Plants were grown for 5 weeks under short day (8h light/16h dark) followed by 1 week long day (16h light/8h dark).

**Supporting Table S1: Subcellular protein marker set.** The table comprises identifiers (ID) of selected marker proteins for cytosol, mitochondria, nucleus, plastid, vacuole and peroxisome.

**Supporting Table S2: Pearson correlated subcellular marker set with total proteome.** All listed proteins for each compartment were identified by Pearson correlation. For assignment to a subcellular compartment, correlation coefficients with marker proteins (Supplemental Table S1) was at least >0.95 in 2 out of 3 replicates and p<0.05 (with Bonferroni correction of multiple comparisons).

**Supporting Table S3: Significant metabolic shifts to mitochondria from a 3-compartment model to a 4-compartment model.** Primary metabolites were shifted significantly from indicated compartments to mitochondria. First column: changes in significances between genotypes at the same time point. Second and third column: Significant changes within genotypes and time points. ‘+’ indicates a shift from two compartments at the same time point, ‘/’ separates two time points.

**Supporting Table S4: Mean values of primary metabolites in 3 compartments and t-Test results.**

**Supporting Table S5: Mean values of primary metabolites in 4 compartm****ents and t-Test results.**
